# Scavenger receptors in host defense: from functional aspects to mode of action

**DOI:** 10.1186/s12964-021-00812-0

**Published:** 2022-01-03

**Authors:** Qamar Taban, Peerzada Tajamul Mumtaz, Khalid Z. Masoodi, Ehtishamul Haq, Syed Mudasir Ahmad

**Affiliations:** 1 Division of Animal Biotechnology, Faculty of Veterinary Sciences and Animal Husbandry, Sher-e- Kashmir University of Agricultural Sciences and Technology – Kashmir, Shuhama, 190006 India; 2 Department of Biotechnology, University of Kashmir, Hazratbal Srinagar, Kashmir India; 3 Department of Nutrition and Health Sciences, University of Nebraska-Lincoln, Lincoln, USA; 4 Division of Plant Biotechnology, Transcriptomics Laboratory, SKUAST-K, Shalimar, India

**Keywords:** Scavenger receptors, Immunity, PAMPs, Signalling pathways, ACE-2

## Abstract

Scavenger receptors belong to a superfamily of proteins that are structurally heterogeneous and encompass the miscellaneous group of transmembrane proteins and soluble secretory extracellular domain. They are functionally diverse as they are involved in various disorders and biological pathways and their major function in innate immunity and homeostasis. Numerous scavenger receptors have been discovered so far and are apportioned in various classes (A-L). Scavenger receptors are documented as pattern recognition receptors and known to act in coordination with other co-receptors such as Toll-like receptors in generating the immune responses against a repertoire of ligands such as microbial pathogens, non-self, intracellular and modified self-molecules through various diverse mechanisms like adhesion, endocytosis and phagocytosis etc. Unlike, most of the scavenger receptors discussed below have both membrane and soluble forms that participate in scavenging; the role of a potential scavenging receptor Angiotensin-Converting Enzyme-2 has also been discussed whereby only its soluble form might participate in preventing the pathogen entry and replication, unlike its membrane-bound form. This review majorly gives an insight on the functional aspect of scavenger receptors in host defence and describes their mode of action extensively in various immune pathways involved with each receptor type.

**Video abstract**

**Supplementary Information:**

The online version contains supplementary material available at 10.1186/s12964-021-00812-0.

## Background

Scavenger receptors (SRs) were shown for the first time on macrophages to function in endocytosis and degradation of modified (acetylated) low-density lipoproteins (LDLs) [1]. SRs are a structurally heterogeneous superfamily of proteins that belong to different classes with very little or no structural resemblance. The only characteristic that designates various classes is their competence to bind mutual ligands. SRs show interactions with modified self-molecules, damage-associated molecular patterns (DAMPs), non-self molecules like preserved pathogen-associated molecular patterns (PAMPs) on microbial pathogens (lipopolysaccharide (LPS) and lipoteichoic acid (LTA)). They also recognize unmodified endogenous proteins, lipoproteins, apoptotic cells and polyionic ligands such as carbohydrates, proteoglycans, cholesterol ester and phospholipids etc. Host cells are effective guardians of the immune response through the expression of complex surveillance systems, including the Pattern Recognition Receptors (PRRs) [2]. Scavenger receptors are membrane-associated pattern recognition receptors (PRRs) [3–5] that act as phagocytic receptors mediating direct non-opsonic uptake of pathogenic microbes and/or their products. SRs may partner with other PRRs like TLRs (Toll-like receptors) or multimolecular complexes on various cell types and participate in diverse functions like signalling other than scavenging. Recognition of pathogens by SRs on antigen-presenting cells leads to inflammatory response followed by phagocytosis, processing of antigens and subsequent presentation on MHC class I and II molecules thus, linking innate and adaptive immune responses [6]. SRs show expression on various cell types that are potential portals of pathogen entry like macrophages, dendritic cells, neutrophils, microglia, B cells, endothelial and epithelial cells [7, 8]. SRs play a significant role in host defence by recognizing countless microbial antigens at the portals of pathogen invasion and activating downstream immune responses to fight and eliminate the pathogens [9].

Due to their functional diversity and involvement in various diseased conditions and immunity-related signalling pathways this review extensively focus upon the emergence of mammalian scavenger receptor as PRRs, their involvement in host defence and mode of action in various immune pathways involved with each receptor type.

### Types of Scavenger receptors

Scavenger receptors are classified based on their nucleotide sequence alignment and protein structure [10, 11]. Each class is divided into subclasses that include members, which share structural features [12]. Based on the current understanding of scavenger receptors and proposed nomenclature, this review discusses 12 classes and their subclasses of mammalian scavenger receptors and one potential scavenger receptor, Angiotensin-converting enzyme-2 (ACE-2).

### Class A

**Fig. 1:**
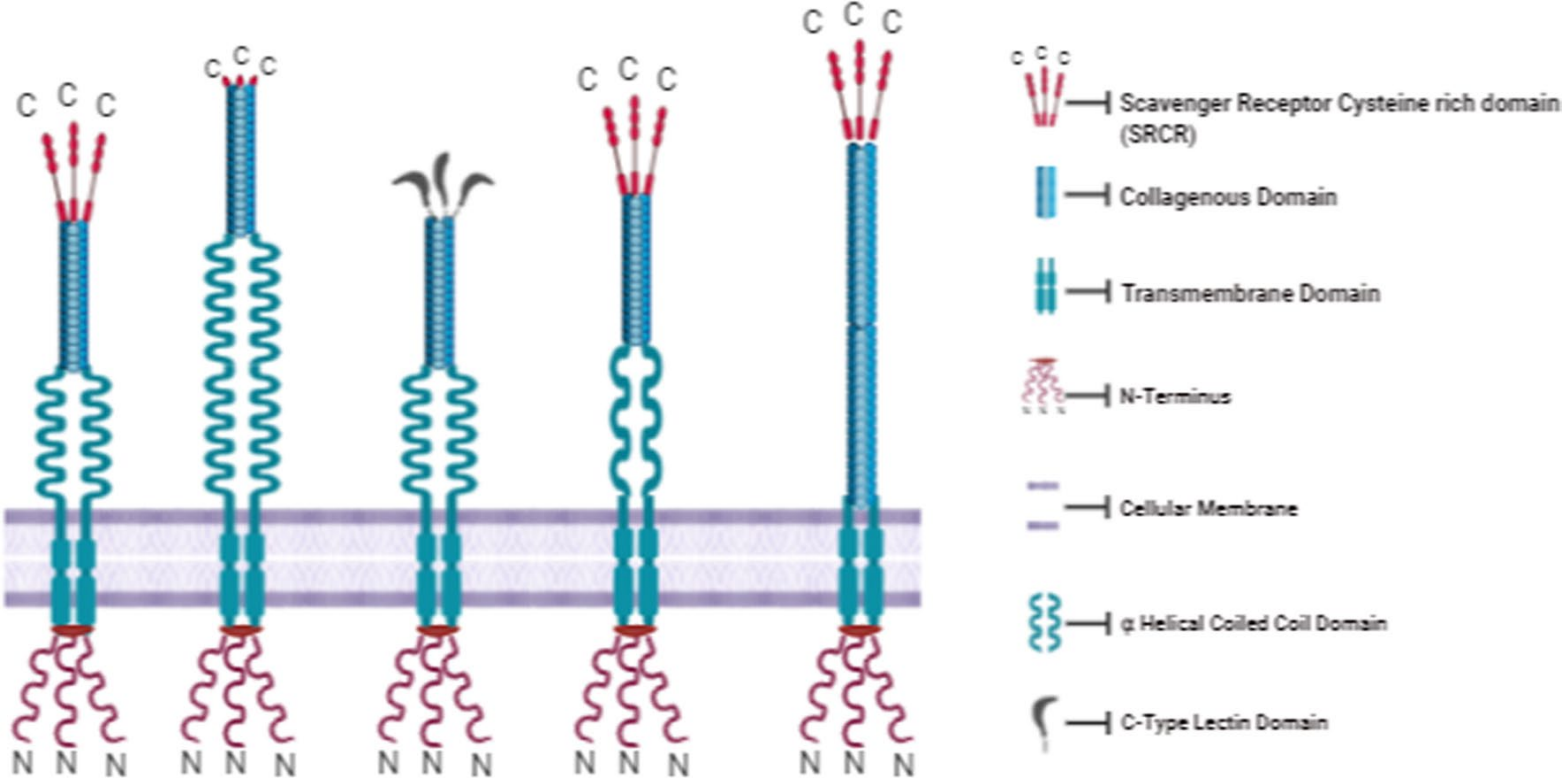
Schematic representation of various members of Types A Scavenger receptor: SR-A1, SR-A3, SR-A4, SR-A5, SR-A6. Various domains are shown in the key within the picture

Class A scavenger receptors have an N-terminus cytoplasmic domain, single transmembrane section and a big extracellular C-terminus part involved in ligand identification. Class A SRs contains a collagen domain, and a type-A cysteine-rich domain (SRCR) or a C-type lectin domain (CLEC). Members include SR-A1 (SCAR-A1 OR MSR1), SR-A3 (SCAR-A3 or CSR1), SR-A4 (SRCL), SR-A5 (SCAR-A5) and SR-A6 (MACRO) (Fig. 1). The linked SRCR domain of SR-A1 facilitates communications with other membrane-bound receptors while the collagen domain is responsible for ligand recognition. SR-A1 binds to lipopolysaccharide (LPS), lipoteichoic acid (LTA) and bacterial CpG DNA. In the presence of LPS, SR-A1 interacts with Toll-like receptor 4 (TLR4) and stimulates NF-κB and inflammatory cytokine production in macrophages in presence of LPS [13]. Also, it binds to the purified lipid A moiety of LPS from *E. coli* where it is involved in the clearance and detoxification of endotoxins [14]. Similarly, SR-A1 also participates in host defence by binding and clearance of LTA and/or gram-positive bacteria from tissues and the circulation such as (*Streptococcus pyogenes, Streptococcus agalactiae, Staphylococcus aureus, Enterococcus hirae and Listeria monocytogenes*) [15]. SR-A1 can facilitate the internalization of *Neisseria meningitides*, *Listeria monocytogenes* and *Staphylococcus aureus* [16–18]. The SRCR domain of SR-A6 mediates the attachment of bacteria and LPS. The positive arginine area that exists outwardly on the exterior of SR-A6 is deficient in the case of SR-A1 [19]. SR-A1 and SR-A6 are also involved in the aberrant dispersal of splenic macrophages if depleted. In mice, deficiency of SR-A1 and SR-A6 results in distorted spleen morphology and low circulating antibody levels (IgM and IgG3) for bacterial polysaccharides. Ligand specificities and structural features suggest that SR-A1 and SR-A6 show functional dissimilarity contrary to a study, which recognizes overlapping, but separate endogenous and microbial ligands, comprising some *N. meningitides* external proteins binding to both of these receptors [20]. SR-A1 can generate an adaptive immune response by stimulating antigen binding, internalization and antigen presentation in alliance with HSP70 members [21]. SR-A1 also cooperates with TLR4 to phagocytize *Escherichia coli*, while SR-A1 and TLR2 collaborate in the phagocytosis of *Staphylococcus aureus* [22]. SR-A6 partners with TLR2 and CD14 in the identification of the *Mycobacterium tuberculosis* glycolipid to produce a pro-inflammatory reaction [23]. Macrophage associated cell surface SR-A6 is inhibited by Herpes simplex virus type 1 (HSV-1) jointly with proteoglycans to facilitate adsorption of the virus to keratinocytes epithelial cells [24]. SR-A1 participates in the internalization via clathrin-dependent endocytosis (CDE) or clathrin-independent endocytosis (CIE) methods and the latter stimulates apoptosis. In antigen-presenting cells (APCs), SR-A1 facilitates internalization and phagocytosis by a lipid raft-dependent method. It was reported that cumulative surface expression of SR-A1 along with its co-receptor MERTK M2 resulted in the engulfment of apoptotic bodies by macrophages [25]. As for SCAR-A3 or CSR1 (cellular stress response protein), its expression could be improved by oxidative stress and functions as a cellular stress response gene to scavenge reactive oxygen species (ROS). A recent study reported SCAR-A3 as the potential prognostic indicator in Hand Foot and Mouth Disease (HFMD) and a boosted expression of SCAR-A3 was observed in severe HFMD patients compared with the control group [26]. SCAR-A4 or SRCL (scavenger receptor with C-type lectin) belongs to collectin family PRRs and the C-terminal domain contains a C-type lectin, instead of an SRCR domain. It generates an immune response on binding to heat-killed *S. aureus, E. coli* and *S. cerevisiae* yeast particles [27] and facilitates non-opsonic phagocytosis of zymosan [28]. SCAR-A5 is a newly recognized class A scavenger receptor that binds to modified LDL particles instead of heat-inactivated *E. coli* and *S. aureus*, signifying its part as a PRR in innate immunity [29]. Increased SCAR-A5 expression causes inactivation of signal transducer and activator of transcription 3 (STAT3), a chief transcriptional watchdog in pro-inflammatory gene expression [30].

### Class B

**Fig. 2:**
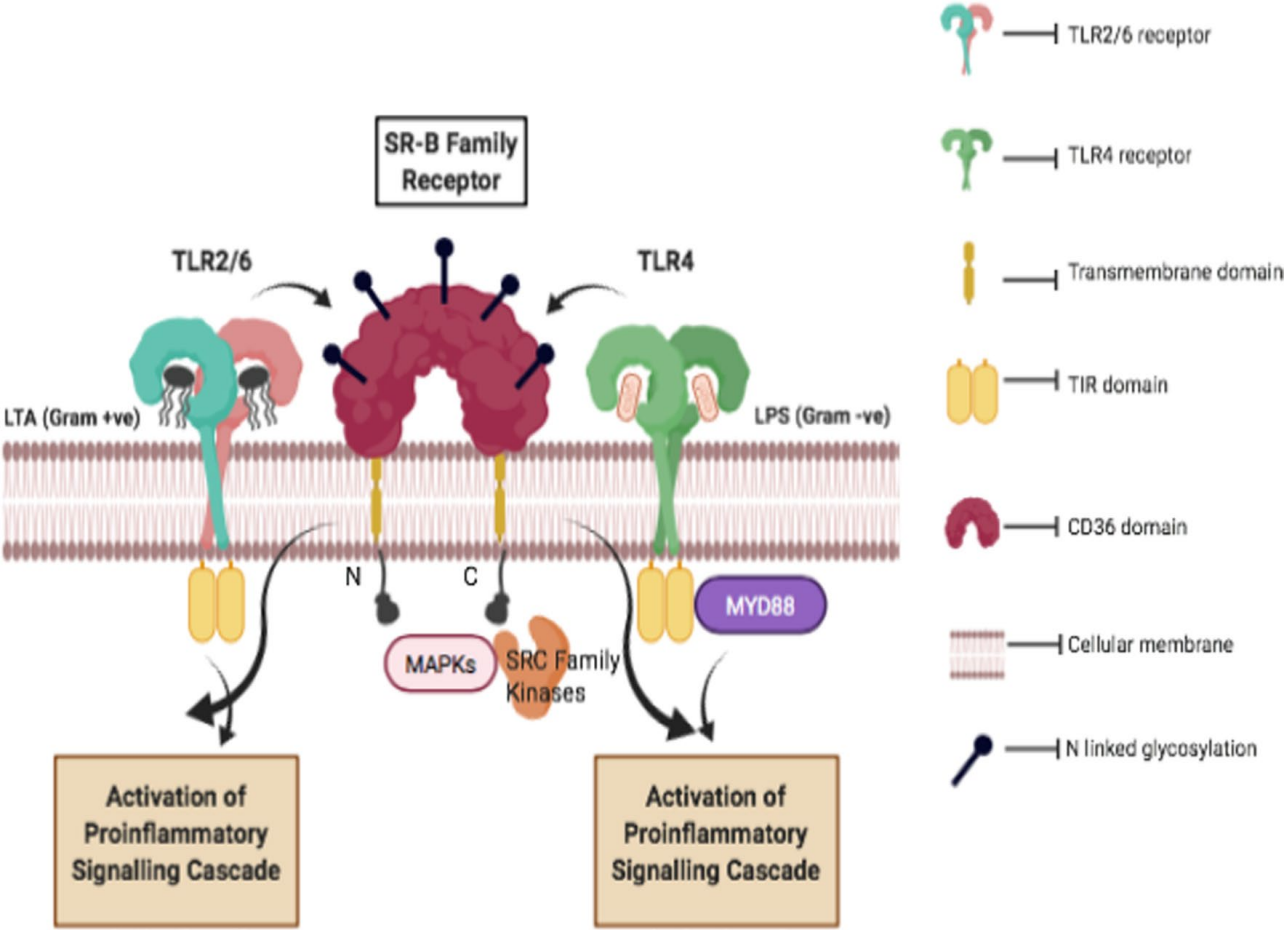
Schematic representation of SR-B receptor class shows interaction with TLR2/6 and TLR4 receptors. TLR2/6 binds to LTA of gram-positive bacteria and TLR4 on interaction with LPS of gram-negative bacteria shows activation of proinflammatory signaling cascade, SRC family kinases and MAPKs

Class B scavenger receptors comprise a conserved CD36 domain (Fig. 2). It comprises three members: SR-B1 (SCAR-B1), LIMP2 (SCAR-B2), and CD36 (SCAR-B3). This class binds to a wide array of ligands like viruses and bacteria, HDL particles and correlates with the amplified danger of infertility, atherosclerosis and reduced natural immunity. CD36 (SCAR-B3) has two transmembrane domains and both its N and C terminus are cytoplasmic. The C-terminal tail might be the spot of signal transduction and links with SRC family kinases, including FYN, YES and LYN [31]. The C-terminus of CD36 holds a CXCX5K motif, which is located on the cytosolic ends of the T cell co-receptors CD4 and CD8 that play a role as a docking place for SRC kinases. CD36 is known to stimulate mitogen-activated protein kinases (MAPKs) and binds with a noticeable array of transmembrane proteins that comprise TLR2, TLR4 TLR6, β1 integrin, β2 integrin, β5 integrin and CD9, CD81. In response to lipoteichoic acid or diacylated lipoproteins, CD36 generates an immune response in association with the TLR2-TLR6 heterodimer complex [32, 33]. The SR-B1 (SCAR-B1) has two splice variants entitled SR-BI and SR-BII and has an indistinguishable loop structure as of CD36. A variety of pathogenic ligands comprising Alexa Fluor 488-labeled live *E. coli* K12, K1, S. *aureus,* S. *typhimurium* and *Listeria monocytogenes* bind and internalize CLA-1 and CAL-2 stably transfected HeLa and HEK293 cells. These cells also bind to and internalize dead bacteria and are involved in its clearance hence their role in infection and sepsis [34]. Another study demonstrated internalization of *E. coli*, LPS, and chaperonin 60 **(**GroEL) in HeLa cells due to overexpression of CLA-1, CLA-2, and CD36 receptors indicating that SR-B1 receptors plays part in pathogen detection and facilitate bacteria-associated inflammation and signalling [35]. Hepatic SR-B1 acts as a critical defensive factor in sepsis thus endorsing hepatic SR-B1 facilitated LPS clearance that delivers a therapeutic approach for sepsis [36]. SR-BI plays role in hepatitis C virus (HCV) internalization and cross-presentation by human dendritic cells (DCs) and may influence the design of HCV vaccines and immunotherapeutic methods [37]. The role of SR-B1 and LOX1 on bronchial epithelial cells (BECs) showed that SRs participate in the in vitro activation of human airway cells triggered by TLR3 ligand, dsRNA and SRs act as transporters, enabling dsRNA entrance and transport to the dsRNA-sensing receptors on BECs [38]. In malaria, SR-B1 acts against host defence for *Plasmodium* infection by stimulating sporozoite penetration in liver cells and consequent intracellular parasite growth [39]. SR-B1 binds to the diverse spectrum of receptors; it binds and recognizes *Mycobacterium tuberculosis *in vitro but is known to play only an insignificant role in anti-mycobacterial immunity in vivo [40]. Overexpressed SR-B1 in epithelial cells of pyometra-affected uteri is potentially involved in endometrial bacterial adhesion and involvement in the pathogenesis of pyometra in general [41]. In our study on milk derived goat mammary epithelial cells (GMECs) we have validated the presence and expression of SR-B1 and its role in *E. coli* infection. Through esiRNA based silencing technique, SCARB1 expression significantly affects the TLR4-MyD88 and TRIF pathway genes following infection with *E.coli.* Also, this receptor is involved in mediating endocytosis of live bacteria in GMECs. Intriguingly, CD36 has a role to play in the clearance of numerous bacterial and protozoan pathogens. A variety of bacteria like *E. coli*, *Klebsiella pneumoniae*, *S. typhimurium*, *S. aureus*, and *Enterococcus faecalis* are phagocytized in CD36 overexpressing HeLa cells via JNK-Mediated Signaling and in association with TLR2/4 [42]. Also, the binding to beta-glucan of *Cryptococcus neoformans* provided evidence of its role in antifungal defence in an experimental mice model in vivo [43]. CD36 deficit presents resistance to mycobacterial infectivity, which is due to decreased intracellular existence of *Mycobacterium* in the *Cd36* -/- macrophages [44]. In goat mammary epithelial cells, CD36 is involved in LPS based pro-inflammatory response and TLR4 mediated *E.* *coli* endocytosis in GMECs [45].

LIMP-2 (lysosome membrane protein 2) or SCAR-B2 is known to be a receptor for enterovirus 71 (EV71) and shows binding with both of its soluble and cell surface forms. LIMP2 is recognized for enterovirus 71 so that its expression is propagated in normal unsusceptible cell lines, development of cytopathic effects. It is also a receptor for development of infection for coxsackievirus A16 (CVA16) (a weak pathogen). Enterovirus 71 belongs to human enterovirus species A and along with CVA16, is recurrently linked with human foot and mouth disease [46]. L929 cells expressing human LIMP-2 infected with coxsackievirus A7, coxsackievirus A14 and coxsackievirus 16 require the receptor for entrance into host cells and the expansion of human foot and mouth disease [47]. Subsequently, during EV71 infection, LIMP-2 along with acidic conditions also function as a receptor for viral binding, virus internalization, viral uncoating and therefore infection efficiency [48].

### Class C

Class C scavenger receptors have only been described in *Drosophila melanogaster* and lacks mammalian counterparts. *Drosophila* SR-CI (dSR-CI) on Schneider 2 cells (derived from a primary culture of late stage *Drosophila* embryos) is expressed as an undefined bacterial PRR for both gram-positive and gram-negative bacteria. Cross-competition experiments and dsRNAi-mediated gene silencing methods have suggested dSR-CI to be a common candidate PRR for *E. coli* and *S. aureus* binding and optimal bacterial phagocytosis by S2 Cells. Additionally, dSR-CII is detected on early embryos and is predicted to be a transmembrane protein with no role in innate immunity at later stages. Also, dSR-CIII and dSR-CIV classes are predicted to be present in secreted soluble form [49].

### Class D

CD68 (named Macrosialin in the mouse) is the only member belonging to class D of scavenger receptors. Class D scavenger receptors contain a mucin-like domain, a proline-rich center, lysosome-associated membrane glycoprotein (LAMP) domains, single transmembrane and a small cytoplasmic tail [50]. CD68 receptor is abundant on immune cells like free monocytes, tissue-specific macrophages in the peritoneum, liver, lungs, spleen, Langerhans cells, and microglia where it scavenges oxLDL binds to lectins, selectins and mediate endocytosis and phagocytosis. CD68 being a macrophage marker is involved in differentiation of hematopoietic cells of the monocyte/macrophage descent. The function of CD68 in antigen presenting and processing is ambiguous. But, the involvement of lysosomal associated membrane proteins (LAMP) in CD68 related activities are highly assumed due to their structural homology. In CD68 knockout mice, phagosome lysosome fusion and overall phagolysosome formation are regulated by LAMP-1/2. Lower levels of CD68 are also expressed in CD4^+^ T lymphocytes, CD19^+^ B lymphocytes, basophils and intestinal neutrophils from patients with inflammatory bowel syndrome. While as in normal mucosal tissue CD68 + neutrophils are absent [51]. In vivo CD68 participates in discriminating M1 and M2 macrophage divergence in association with transcription factor markers such as pSTAT1, CMAF and RBP-J [52] and association of TLR4 in (microglial cell) macrophages in brain tissue in response to stimuli like LPS and IFN-*γ* upregulates the CD68 expression significantly [53]. CD68 plays a role in host resistance by preventing the uptake of malarial sporozoite in liver tissue macrophages hence acting as a potential receptor for malarial pathogen [54]. Contrary to this in small intestinal epithelial cells, CD68 was reported to be putatively involved in antigen processing and presentation together with other factors in the processing of intestinal pathogens [55]. In summary, the participation of CD68 in immunity and inflammation is still ambiguous and needs further validation.

### Class E

**Fig. 3:**
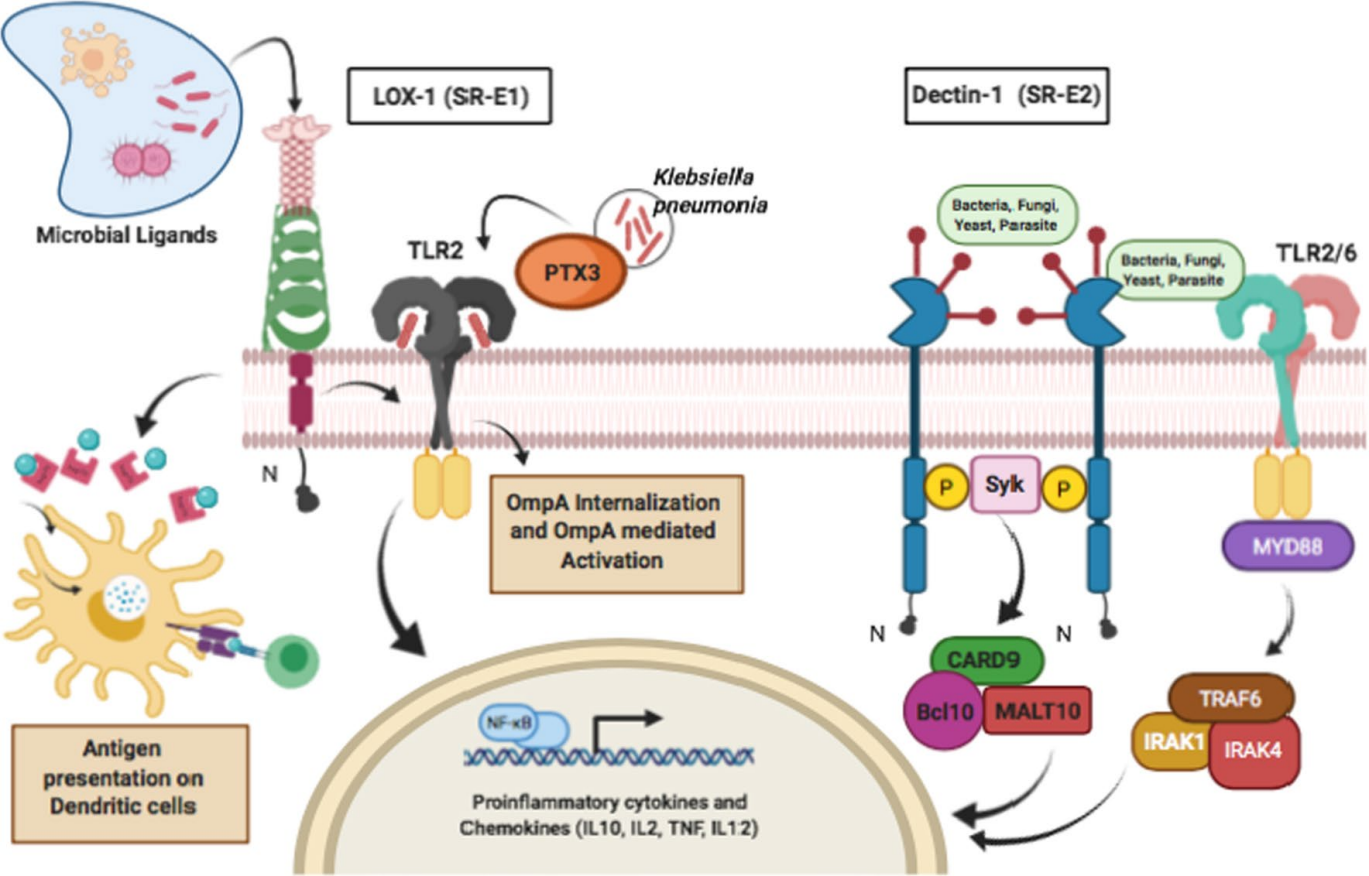
(**a**) LOX-1 on interaction with TLR2 on infection with PTX3 of Klebsiella Pneumonia shows OmpA internalization and stimulation of proinflammatory response. On binding to diverse microbial ligands it participates in antigen presentation on MHC class-I of dendritic cells in association with heat-shock protein HSP70. (**b**) Dectin-1 on binding to diverse antigens dimerize and activate CARD9, Bcl10, and MALT10, while on binding to TLR2/6 activates TRAF6, IRAK1, and IRAK4 leading to activation of proinflammatory cytokines and chemokines

Class E scavenger receptors belong to the NK cell C-type lectin-like (CLEC) receptor family. It has four members: SR-E1 (LOX-1) (Fig. 3a), SR-E2 (Dectin-1), SR-E3 (MRC1) and SR-E4 (ASGPR1). The SR-E1 is also called lectin-like oxidized low-density lipoprotein receptor (LOX-1). Human SR-E1 has an N-cytoplasmic region, a transmembrane region, an extracellular coiled-coil ‘neck’ region and a C-type lectin-like domain. SR-E1 binds diverse ligands like apoptotic cells, gram-positive, gram-negative bacteria and acute phase C-reactive proteins. SR-E1 participates in antigen presentation on MHC class-I of dendritic cells in association with HSP70 [56]. SR-E1 also mediates signal transduction that triggers an important feature of pro-inflammatory response in immune and vascular cells i.e., NF-κB activation [57]. It acts as an intermediate between NF- κB and its targets. In Chinese hamster ovary-K1 (CHO-K1) cells, stably expressing LOX-1 can bind FITC-labeled *S. aureus* and *E. coli* in both static and non-static conditions, and bovine aortic endothelial cells (BAEC) also bind to labelled *S. aureus* that is supported by the fact that binding was repressed with poly (I) and an anti-LOX-1 mAb [58]. Knockout of LOX-1 decreased pro-inflammatory response, reduced inflammation during sepsis, lung oedema, stopped neutrophil overreaction, and amplified neutrophil employment to infection sites in a murine model of polymicrobial sepsis. Thus, indicating that SR-E1 is a significant intermediary of intracellular signalling during infection and promotes immune suppression if absent [59]. In the brain abscess model, TLR2-dependent signals affect the degree of SR-E1 induction, suggesting possible cross-talk amongst TLRs and SRs. Both SR-A1 and SR-E1 together generate an antibacterial immune response in the CNS parenchyma [56]. Also, TLR2 activation is triggered when SR-E1 together with SR-F1 binds to outer membrane protein A (OmpA) of *Enterobacteriaceae (Klebsiella pneumonia)* and thus controls many phases of the innate immune response [60].

SR-E2 or Dectin-1 being an innate immunity PRR is expressed principally on macrophages, DCs, and neutrophils. This receptor mediates both the internalization and cellular responses of various bacteria, fungi and parasites through unique processes [61]. Dectin-1 stimulates diversity of cellular reactions like phagocytosis; cytokine production and the respiratory burst via Syk/CARD9 dependent and Syk- independent signalling pathways [62]. Dentin-1 recognizes unidentified endogenous ligands on CD4 + and CD8 + T cells hence acting as a co-stimulatory molecule through an unknown response. Due to its prevalence on DCs and macrophages of medullary areas of the thymus, it functions in thymocyte growth and its expression on CD11c ( +) splenic DCs in areas of the spleen and lymph nodes suggests it to act as a co-receptor for triggering T cells [63]. Dectin-1 plays a major role against the systemic *Candida glabrata* challenge. Splenocytes were collected from infected dectin-1-deficient and wild-type mice and the levels of TNF- α, IL-6, IFN-ϒ and IL-17 in supernatant indicated lower Th cell responses. Also, dectin-1-and dectin-2 deficient mice showed considerably increased fungal loads while dectin-1 renders the host sensitive to *C. glabrata* infection, unlike dectin-2 [64] (Fig. 3b).

SR-E3 or MRC1, the human mannose receptor (CD206) is another transmembrane glycoprotein belonging to this class. Most tissue macrophages, DCs and selected lymphatic or liver endothelial cells express it predominantly. It is involved in phagocytosis of mannosylated glycoproteins, or receptor-mediated antigen presentation. As a homeostatic PRR on macrophages, it binds to high mannose *N*-linked glycoproteins on the surface of pathogens, pituitary hormones in the circulation and scavenges via phagocytosis and lysosomal degradation. SR-E3 after participation in recognizing and processing antigenic bacteria help in the removal of myeloperoxidases that are released by the pathogenic bacteria to prevent complement activation and damage to host tissue. CD206 binds to a variety of pathogens like M*. tuberculosis,* S*. pneumonia, Yersinia pestis, Candida albicans, Pneumocystis carinii, Cryptococcus neoformans*, HIV, influenza virus, dengue virus, and *Leishmania* species. MR-mediated uptake by macrophages in tissues during infection is a striking method for effective and targeted delivery of drug transporters such as liposomes, microparticles, nanoparticles and dendrimers for infectious diseases like tuberculosis and also for cancer imaging, diagnosis and therapy [65]. SR-E3 is expressed on dendritic epidermal cells in a condition called atopic dermatitis where it acts as a differentiation marker of immature monocyte-derived DCs [66] and in COPD (severe chronic obstructive pulmonary disease) overexpression of SR-E3 along with other CD markers on alveolar macrophages function in COPD pathogenesis [67]. In hepatitis B virus mouse model F4/80^+^SR-E3^+^CD80^lo/+^ hepatic macrophages endorse the immunosuppressive action of regulatory T cells thus offering novel understandings into the immunomodulation in HBV infection [68].

SR-E4 or asialoglycoprotein receptor 1 (ASGPR1) also designated the Ashwell receptor is found on the surface of hepatocytes that recognize, internalize and transport glycoproteins deficient in terminal sialic acid residues and those which have galactose or N-acetylgalactosamine residues via the route of receptor-mediated endocytosis. SR-E4 binds to a range of clinically essential plasma proteins like transferrin, IgA, apoptotic cells, fibronectin, alkaline phosphatase and many immune cells. During liver diseases impaired SR-E4 receptor is related to the increased pro-inflammatory release of TNF-α and IL-6 in kupffer cells [69]. Additionally, when mice deficient in functional hepatic SR-E4 (receptor-deficient, RD), and wild-type (WT) controls were intravenously administered with mitogens, the former displayed increased proinflammatory cytokine expression, caspase activation and buildup of CD8 + T cells versus normal WT mice. Thus, deficiency of this receptor may lead to liver diseases as it has protective effects against T cell-mediated hepatitis [70]. Similarly, in SR-E4 knockout hepatitis E virus-infected PLC/PRF/5 cells various assays revealed direct attachment of ASGR1 and ASGR2 to ORF2 protein of virus and participation in regulating the viral attachment and internalization steps and not in viral emancipation. Also, HeLa cell lines stably expressing SR-E4 scavenger receptors demonstrated amplified virus-binding competence [71].

### Class F

**Fig. 4:**
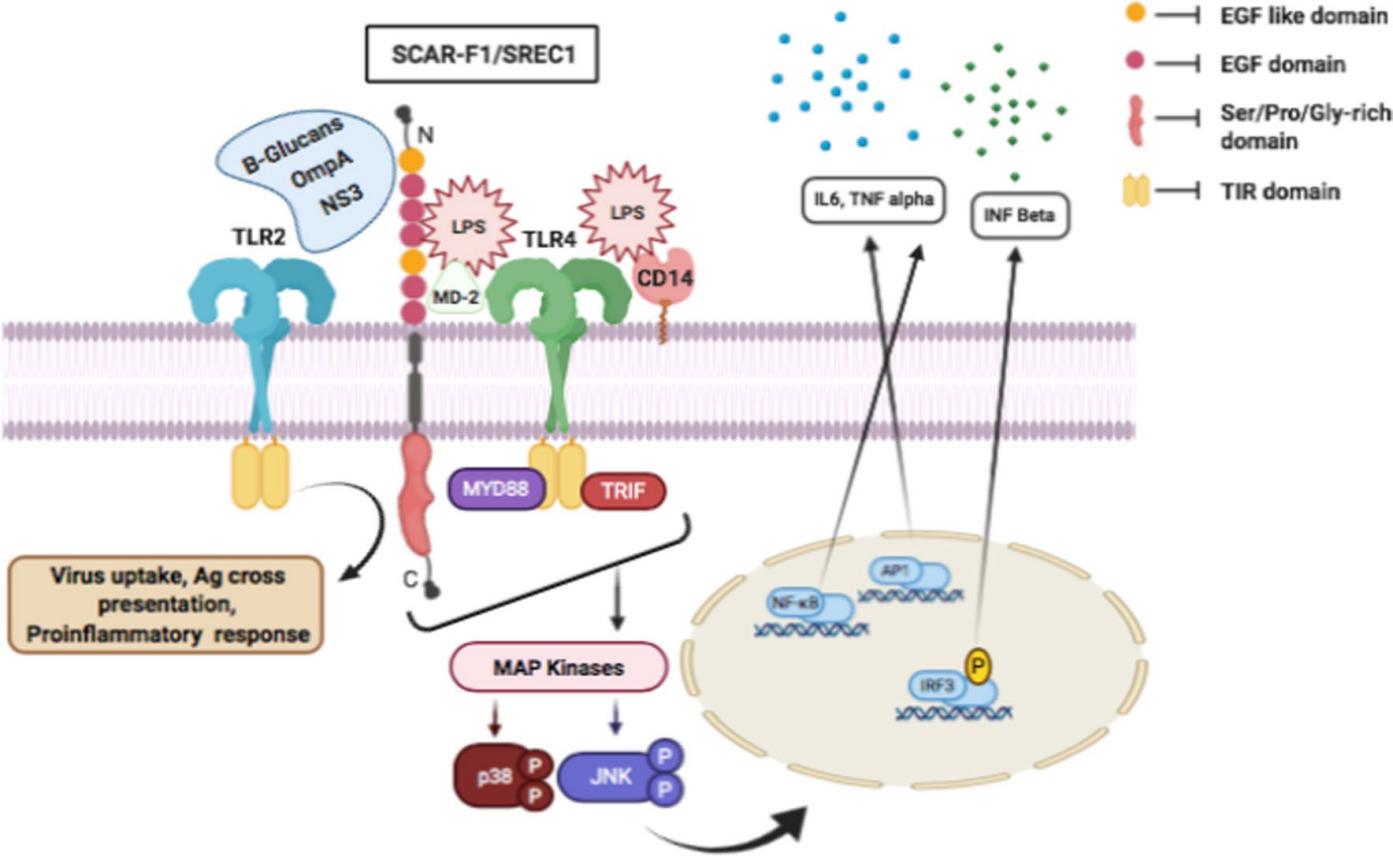
SCAR-F1 on binding to diverse ligands like B-glucans, OmpA, NS3 in presence of TLR2 activates proinflammatory response, Antigen cross-presentation and viral uptake. On interaction with TLR4 in presence of LPS it activates MAP kinases following activation of various transcriptional factors like p38 and JNK that mediate extracellular release of IL6, TNF-α and INF-β

Class F scavenger receptors have three members: SREC1 (SCAR-F1), SREC2 (SCAR-F2) and SCAR-F3 (also called MEGF10) and have epidermal growth factor (EGF) and EGF-like domains. One of the distinct structural properties of these receptors is that they lack visible signalling motifs on short cytosolic ends. They also have a higher inclination to oligomerize and bind large, multivalent ligands. SCAR-F1 is a membrane-bound receptor with EGF-like domains on the outside and unusually extended proline- and serine-rich cytoplasmic extensions. SCAR-F1 binds to a variety of pathogens, both exogenous and endogenous. It binds to fungal pathogens in a β-glucan dependent approach and facilitates host defence alongside *Candida albicans* and *Cryptococcus neoformans.* SCAR-F1 along with SCAR-B3 mediated cytokine production and innate immunity in response to fungal infections [72]. Also, SCAR-F1 through an endocytic receptor in co-operation with TLR2 binds to non-structural protein 3 (NS3) of hepatitis C virus and participate in virus uptake and cross-presentation [43]. SCAR-F1 in association with TLR4 leads to LPS induced pro-infalmmatory response through NF-kB and P kinase pathways on RAW and HEK 293 cells and function in the endocytosis of peptides and antigen presentation [73]. SCAR-F1 is present on DCs and helps in the removal of apoptotic cells in association with C1q/phosphatidylserine complexes. Deficiency of SCAR-F1 has been shown to impair efferocytosis in vitro and in vivo and trigger systemic lupus erythematosus, an autoimmune disorder in SCAR-F1 deficient mice [74] (Fig. 4).

SCAR-F2 is originally recognized on endothelial cells and is expressed by macrophages as well but its scavenging function is yet to be reported. It favourably forms heterodimers with SCAR-F1 in *trans* and these heterodimers lack SR activities losing the competence to mediate ligand recognition [75].

MEGF10 (Multiple EGF-like domains 10) or SCAR-F3 is the third newest member in this group that was recently reported to express on brain macrophages (myosatellite and astrocytes). It plays a role as a receptor for binding to a complement protein called C1Q and is hence involved in apoptotic cell clearance in the mouse cerebellum and deficiency of MEGF10 in mice showed paucity in the apoptotic cells clearance in the mammalian brain [76]. MEGF10 is reported as an astrocytic phagocytic receptor for neuronal debris and unnecessary synapses in ischemic injured and developing brain [77]. MEGF10 is an ortholog of *Drosophila* Draper [78] and *C. elegans* CED-1 [79] that help to mediate axon pruning by glial cells in flies and phagocytosis of apoptotic cells in worms. It is a critical protein in the synapse remodeling underlying neural circuit refinement and has important implications for understanding learning and memory as well as neurological disease processes. Developing mice deficient in MEGF10 receptor fail to normally refine their retinogeniculate connections and retain excess functional synapses [80].

### Class G

**Fig. 5:**
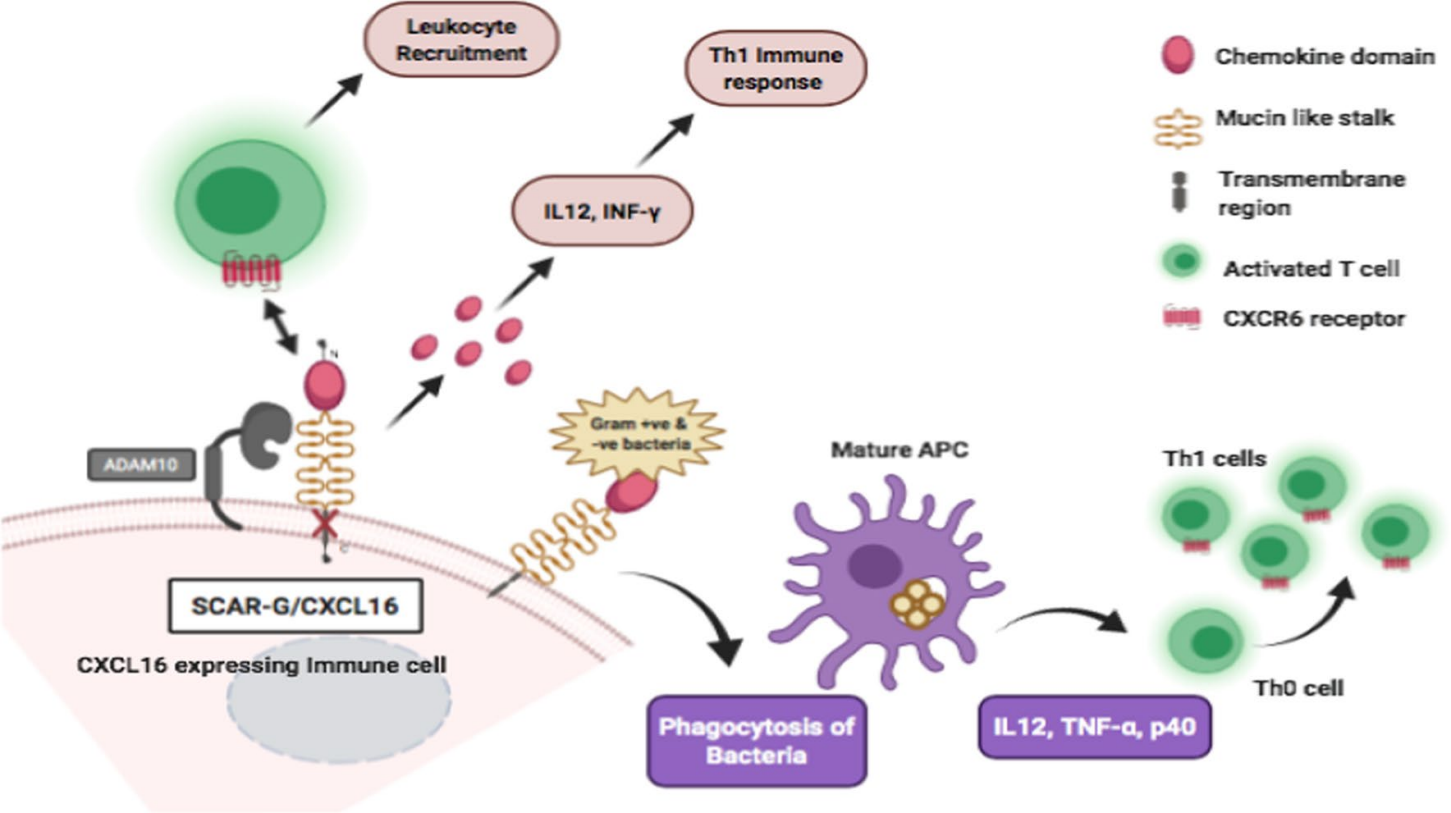
SCAR-G on immune cell activates various immune related pathways like Th1 immune response via activation of IL12 and IFN-γ. On binding to gram-negative and gram-positive bacteria this receptor activates phagocytosis of bacteria into mature APCs following activation of IL12, TNF-α and p40 that helps to convert Th0 into Th1 cells

Chemokine 16 (CXCL16) is a single receptor in this class that has a CXC-chemokine domain with conserved arginine residues (Fig. 5). It is also called SR-PSOX (scavenger receptor for phosphatidylserine and oxidized LDL) due to their amino acid sequence similarity. It was primarily recognized in human monocytic cell line THP-1 as a receptor for scavenging and delivery of oxLDL and as a chemoattractant for stimulated T cells and bone marrow plasma cells via receptor interaction with CXC-chemokine receptor 6 (CXCR6). It is likewise expressed on various immune cells like DCs, macrophages, [81] smooth muscle cells and endothelial cells. Novel CXCL16 on the surface of APCs function as an adhesion molecule that can be converted into a soluble form through proteolytic degradation of transmembrane CXCL16 mediated through (A Disintegrin and metalloproteinase domain-containing protein 10) ADAM10, which acts as a CXCL16 sheddase. The soluble chemokine, SR-PSOX/CXCL16 is interferon-regulated that triggers the CXCR6 receptor expressed by T cells, natural killer T cells [82] and a range of CXCR6 ( +) leukocytes. In chronic inflammation, a characteristic of inflammatory bowel disease is that the serum concentrations of soluble SR-PSOX/CXCL16 are elevated in patients and that this soluble cytokine triggers phagocytosis of bacterial pathogens as well as Th 1 immune response through the production of IL 12, TNF- α and INF γ [83]. The deficiency of SR-PSOX/CXCL16 however, results in a reduced number of natural killer T cells in the liver and diminished release of cytokines like IFN- γ and IL-4 thus explaining its critical role in Th1 immune response [84]. Also, SR‐PSOX/CXC ligand CXCL16 not only attracts but also facilitates the strong adhesion of CXCL16 expressing macrophages and DCs with CXCR6 expressing activated T cells and natural killer T cells [85]. In the liver, a specific NK cell population (liver-resident NK) is critical for local innate immunity that expresses a unique repertoire of chemokine receptors including CXCR6 that regulates selective movement in reaction to the chemotactic stimuli [86].

### Class H

Class H scavenger receptors are transmembrane protein receptors with fasciclin, EGF-like, and lamin-type EGF-like domains comprising scavenger receptor 1, FEEL-1 (also called stabilin-1 and CLEVER1) and FEEL-2 (also called stabilin-2 and HARE). Both are structurally homologous and display analogous domain organization in extracellular regions.

FEEL-1/Stablin-1 is principally expressed on macrophages, mononuclear cells, hematopoietic stem cells, and endothelial cells. Its expression is inducible in reaction to diverse proinflammatory stimuli. Stablin-1 as a leucocyte adhesion molecule is involved in regulating lymphocyte recirculation and trans movement to inflammation sites in vitro [87]. Another study by Karikoshi et al*.* confirmed that stablin-1 participates in the movement of T cells and B cells across HEVs in vivo and blockade of this receptor inhibited the relocation of blood monocytes and lymphocytes into the spot of infection [88]. Stabilin-1 binds a broad spectrum of ligands, such as modified LDLs, apoptotic cells and microparticles from gram-positive and negative bacteria. Stablin-1 provides a defence mechanism to cells against bacteria and the direct interaction of stablin-1 and S. *aureus* is confirmed by using a blocking antibody against the transiently expressing receptor on CHO-1 cells [89].

FEEL-2/Stablin-2 has a conventional NPxY-like endocytic motif in the cytoplasmic region and like stablin-1 it is expressed on HS endothelial cells facilitating lymphocyte trafficking to the liver sinusoidal endothelium [90] and binding a variety of ligands such as acLDLs, heparin, apoptotic, necrotic cells and microparticles of gram-positive and negative bacteria. Stablin-2 also participates in regulating lymphocyte recirculation and migration to the liver sinusoidal endothelium via interaction with fasciclin 1 (FAS1) domains of stabilin-2 with lymphocyte expressed αMβ2 integrin. Future findings are necessitated to examine and authenticate the possible function of stabilin-2 in leukocyte trafficking to other tissues like the spleen and lymph nodes [91].

### Class I

Class I receptors are the CD163 family of molecules that are categorized by the presence of numerous group B SRCR in their extracellular region. SCAR-I1 (also known as CD163A) is a transmembrane type 1-membrane glycoprotein with nine SRCR domains that are predominantly expressed in monocytes and macrophages acting as an endocytic receptor for haptoglobin-haemoglobin complexes to endorse the clearance of plasma haemoglobin. It was also called the ‘haemoglobin scavenger receptor’ due to its part as a haemoglobin receptor. It contributes to functions like apoptotic cell sequestration, clearance and inactivation of pro-inflammatory cytokine and TNF‐related weak inducer of apoptosis (TWEAK) [92]. Like scavenger receptor SR-PSOX, SCAR-I1 is highly predisposed to cleavage by exofacial proteases and exists in soluble forms in plasma thus acting as a potential biomarker for infection and autoimmune diseases [93]. However, the proteolytic products function contrarily than the precursor receptor as the soluble form can counteract the growth of pathogens by acting as an iron chelator [94]. CD163A in host–pathogen interactions deliver host protection through its function as a macrophage receptor for gram-negative and positive bacteria. Its expression on human monocytes also provides defence through bacteria-induced proinflammatory cytokine production confirmed through using antagonistic antibodies against CD163A. It also has an immunomodulatory function as it activates intracellular protein tyrosine kinase-dependent signalling secretion of IL-6 and IL-10 [95]. CD163-L1 (also known as CD163B) is another member of this class with 12 SRCR domains. The cytoplasmic splice variants recognized so far are the full-tail length variant (CD163-L1α) and the short-tail variant (CD163-L1β). The subcellular location of these two variants in HEK293 cells differs as the former is an exterior receptor and the latter is in the intracellular section [96]. It is highly expressed in co-localization with CD163 in various types of macrophages like in alveolar macrophages, glia, and kupffer cells. Its involvement in the differentiation of monocytes into macrophages is dependent on various exogenous stimuli; M-CSF, IL6 and IL10 but is repressed by the cytokines such as IL-4, IL-13, TNF-α, LPS/IFN-γ [97]. Being an endocytic receptor, it is internalized through a clathrin-mediated pathway unlike other members of this group, CD163 and CD5 and also does show the same ligand preferences as of CD163 ligands such as the haptoglobin–haemoglobin complex or numerous bacteria.

The third CD163 family member is CD163c-a (SCART1) or CD5 with five SRCR domains. Two isoforms differ in the number of SRCR domains, one with two domains and another with four SRCR domains. SCART1 and SCART2 are the isoforms of this class expressed on mice γδ T cells, lymph node, trachea, and lungs [98] and CD163 and SCART1 genes are expressed in bovine γδ T cells, monocytes, lymph node, lungs, and intestinal lymphocytes [99]. These SRCR families of receptors exist in both bound and soluble forms, whereby the membrane-bound form is involved in ligand binding. CD5 binds to numerous fungal cells such as *Schizosaccharomyces pombe*, *Candida albicans*, and *Cryptococcus neoformans* through its ectodomain and purified zymosan but not gram-negative or positive bacteria or purified LPS, LTA or peptidoglycans components. CD5 binds to fungal particles through conserved fungal components on the surface called β-glucans and trigger phosphorylation of MEK and ERK1/2 and thus triggers MAPK signalling cascade. This interaction further results in the significant release of cytokine IL-8 from HEK293 cells expressing CD5. Whether CD5 binds to microbial ligands in association with TLRs and participate in adaptive immune responses needs further investigation [100]. The fourth human CD163 family molecule is CD163c-b (SCART2) or CD6 that has high structural and functional homology with CD5 ectodomain. Both CD5 and CD6 are lymphocytic receptors found on T and B cells. CD6 is differentially expressed on CD56 NK cell subpopulation and trigger cytokines (INF-γ and TNF-α) and chemokines such as IP-10 and CXCL1 [101]. In Sjögren's syndrome, CD166 is highly expressed on epithelial cells. Unlike CD5, both soluble and membrane-bound forms of CD6 bind to gram-negative and positive bacteria while its soluble form shows less affinity to fungal species (binds to saprophytic but not pathogenic). CD6 binds to both LPS and LTA components in presence of calcium and activate the MAPK signalling cascade [102].

### Class J

**Fig. 6:**
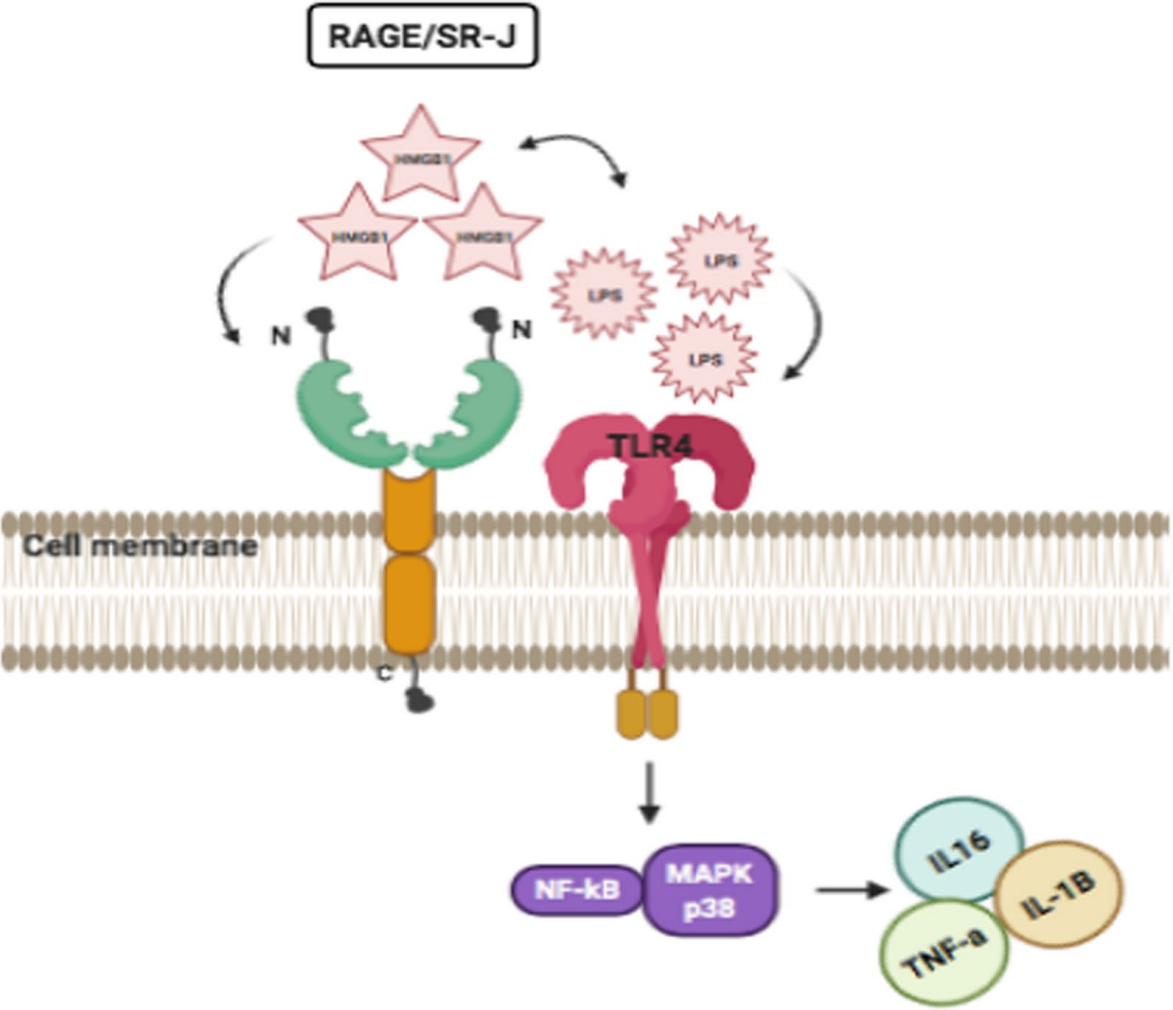
SR-J/RAGE interacts with TLR4 receptor in response to synergistic interaction between HMGB1-LPS complex in activating the macrophages through phosphorylation of MAPK p38 and activation of NF-κB following release to proinflammatory molecules like IL16, IL1-β and TNF-α

RAGE (receptor for advanced glycation end-products) is the only member of class J of scavenger receptors belonging to the Ig superfamily of cell surface molecules (Fig. 6). The ectodomain of this receptor is known to show various ligand interactions with amyloid-β-protein, HMGB1, and microbial PAMPs and DAMPs [103]. As a PRR it is involved in chronic inflammation and immunity, share common ligands and pathways with TLRs thus cooperating synergistically. RAGE interacts with TLR4/2 associated adaptor proteins (TIRAP and MyD88) to activate downstream signalling pathways [104]. HMGB1, a ligand of RAGE works in cooperation with LPS in triggering the macrophages through phosphorylation of MAPK p38 and activation of NF-kB as seen in experimentally induced arthritis in mice [105]. While HMGB1-LPS complexes use TLR4, the HMGB1-Pam_3_CSK_4_ complexes use TLR2. RAGE-HMGB1 interactions are stabilized by heparin sulfate that readily forms a complex with RAGE at the cell surface before binding to HMGB1 [106]. S100 protein family members also interact with RAGE triggering immune responses in cooperation with TLR4 and activation of p38 MAPK, NF-κB and downstream signalling molecules [107]. Despite evidence that S100A8/A9 complex also interacts with TLR4 directly via MD2, [108] it is yet to be investigated if glycans expressed on TLR4 also mediate binding between the S100A8/A9 and TLR4. Also, in vitro analysis indicates that RAGE has a higher affinity with S100A8/A9 than TLR4, whereby the former interaction is linked with inflammation-mediated carcinogenesis and the latter with autoimmune disorders and infection [109]. Direct interaction of RAGE with LPS molecule was also determined through competition assay with another RAGE ligand, AGE-BSA [110] and produced comparable immune reactions as that seen with TLR4 binding in the in vitro and in vivo*.* Unlike, HMGB1–RAGE interactions in synergy with LPS and TLR4 has been demonstrated, the RAGE-TLR4 interactions in response to LPS or whole bacteria are still ambiguous. Like membrane-bound forms, the soluble form of RAGE called sRAGE also functions in various processes and disease pathogenesis [111].

### Class K

The only receptor of class K is CD44 and is a hyaluronan (HA) receptor that shows ligand binding with proteoglycans, growth factors, cytokines, and matrix metalloproteinase through its extracellular domains. ADAM10, ADAM17, and MMP14 act as sheddase of membrane bound CD44 in various tumour cells lines. The external ectodomain cleavage product of this receptor is biologically active [112] and it participates in intracellular signalling through the Src family of kinases such as Src, Lck, Fyn and Lyn and activates small Rho GTPases. CD44 has a wide ligand spectrum and interactions with a diverse range of receptors thereby activating multiple signalling pathways. The most important interactions in the context of immunity are with TLRs. In acute pulmonary infection, CD44 prevents overstated inflammatory responses to LPS. Intratracheal LPS treatment in CD44-/- mice show a marked increase in NF-κB, inflammatory cell recruitment, raised chemokine expression in lung tissue in vivo and reduced induction of the negative regulators of TLR4 signalling pathways [113]. A direct association between CD44 and TLR2 was shown in a study that demonstrates, on stimulation with TLR2 ligand, zymosan, CD44 promoted NF-κB deactivation, suppression of proinflammatory cytokine and that CD44 and TLR2 function together in diminishing TLR-mediated inflammation in CD44 + / + macrophages derived from mice as compared to CD44-/-macrophages [114]. CD44 also functions in response with hyaluronan and LPS in association with TLR4 against the septic response to LPS and show-decreased serum IL-6 and TNFα in CD + / + mice [115]. However, in osteoarthritis, activation of TLR2 and TLR4 induce IL-1β and TNF-α release that notably increased CD44 gene expression and protein concentrations in human macrophages, whereas blocking CD44 with anti-CD44 Ab or HA show opposite results [116]. CD44 also aids in host defence against Group A *Streptococcus* (GAS) through the interaction of CD44 to capsular HA polysaccharide of the bacteria. In transgenic mice expressing a CD44-antisense transgene, no bacteria were internalized by macrophages, thus adding to the fact that CD44 functions as a phagocytic receptor via HA signalling. In macrophages, the molecular mass of HA also determines if the bacteria undergo phagocytosis or not. While degradation of HA with protease, hyaluronidase augmented internalization of GAS by macrophages [117]. CD44-HA interactions are also responsible for progression towards gastric cancer after *Helicobacter pylori* infection whereby a cascade is triggered, which leads to degeneration of parietal cells followed by neoplasia [118]. Similarly in pneumonia, CD44 plays a positive but opposite role in the advancement of infection instigated by *Escherichia coli* and Streptococcus species*.* Unlike E.*coli* based pneumonia, S. *pneumonia* and *Klebsiella pneumonia* induced pneumonia shows decreased CD44–HA-mediated signalling and downstream activation of inflammatory pathways [119]. Overall these reports indicate CD44 prolong bacterial infections by decreasing lung inflammations and increasing bacterial diffusion to other locations. In viral diseases such as HIV and hepatitis C, CD44 plays the opposite role in infection. In HIV, the virus acquires the CD44 molecules from the host and decreases the activation of blood mononuclear cells, CD4 ( +) T cells (by not triggering protein kinase C-α release), and M7-Lue cells in presence of endogenous HA which shows a defensive role in HIV by interfering with CD44-HA interactions [120]. While as in hepatitis C, the CD44 expression is amplified in infected cells with HCV when induced with HA that increases IP-10 (gamma interferon-inducible protein 10) expression via CD44–TLR2–MyD88 interactions [121].

### Class L

Class L has two receptors named: SR-L1 (also called LDLR-related protein 1 (LRP1)) or CD91 and SR-L2 (LRP2 or Megalin). They belong to the LDLR gene family and SR-L1 is the one most studied so far. SR-L1 functions uniquely as a scavenger receptor thus scavenging the extracellular ligands or bioactive compounds (cross-presentation) that come in its contact as well as an extracellular sensor that senses the same ligands and transfer the signal to the cell's interior for activation of classical signal transduction pathways (costimulation). While doing so it is known to bind over 100 diverse ligands whose functions and interactions with other co-receptors and signal transducers mainly remain unknown. One of the important ligands is defensins that are endogenous peptides with antimicrobial action alongside a broad spectrum of pathogens including bacteria, fungi, viruses, and many parasites. SR-L1 expressed on dendritic cells is upregulated by human defensins, HNP-1 alpha defensin or HBD-1 and thus show the existence of an autocrine loop [122]. SR-L1 act as a receptor for heat shock proteins such as gp96, hsp90, hsp70, and calreticulin on APCs (Macrophages, T cells and DCs) and is one of the potential receptors that mediate APC- HSP interactions. It conducts signal to APCs for their activation in presence of immunogenic HSPs followed by stimulation of NF-*κ*B and p38 MAPK and release of TNF-*α*, IL-1*β*, IL-6, IL-12, and GM-CSF [123] and costimulatory and maturation markers like CD80, CD86, CD40, and MHC II [124]. The release of proinflammatory cytokines triggers chronic inflammation during increased levels of hsp70 in synovial fluid from swollen joints of rheumatoid arthritis patients that triggers autoimmunity [125]. However, whether CD91 functions along with TLRs on immune cells in inflammatory processes needs more validation.

Megalin or SR-L2 is another endocytic receptor that belongs to this class, which is expressed on various cells. Megalin is expressed at the blood–brain barrier and lack of this receptor leads to neuroinflammatory processes and impaired neurogenesis by triggering the discharge of pro-inflammatory cytokines such as IL-1β, IL-6 and TNF-α on activation of microglial and astroglial cells and attenuating the suppressor of cytokine signalling-3 (SOCS3) in cortical and hippocampal regions [126].

### Angiotensin-converting Enzyme 2 (ACE-2): a scavenger receptor?

**Fig. 7:**
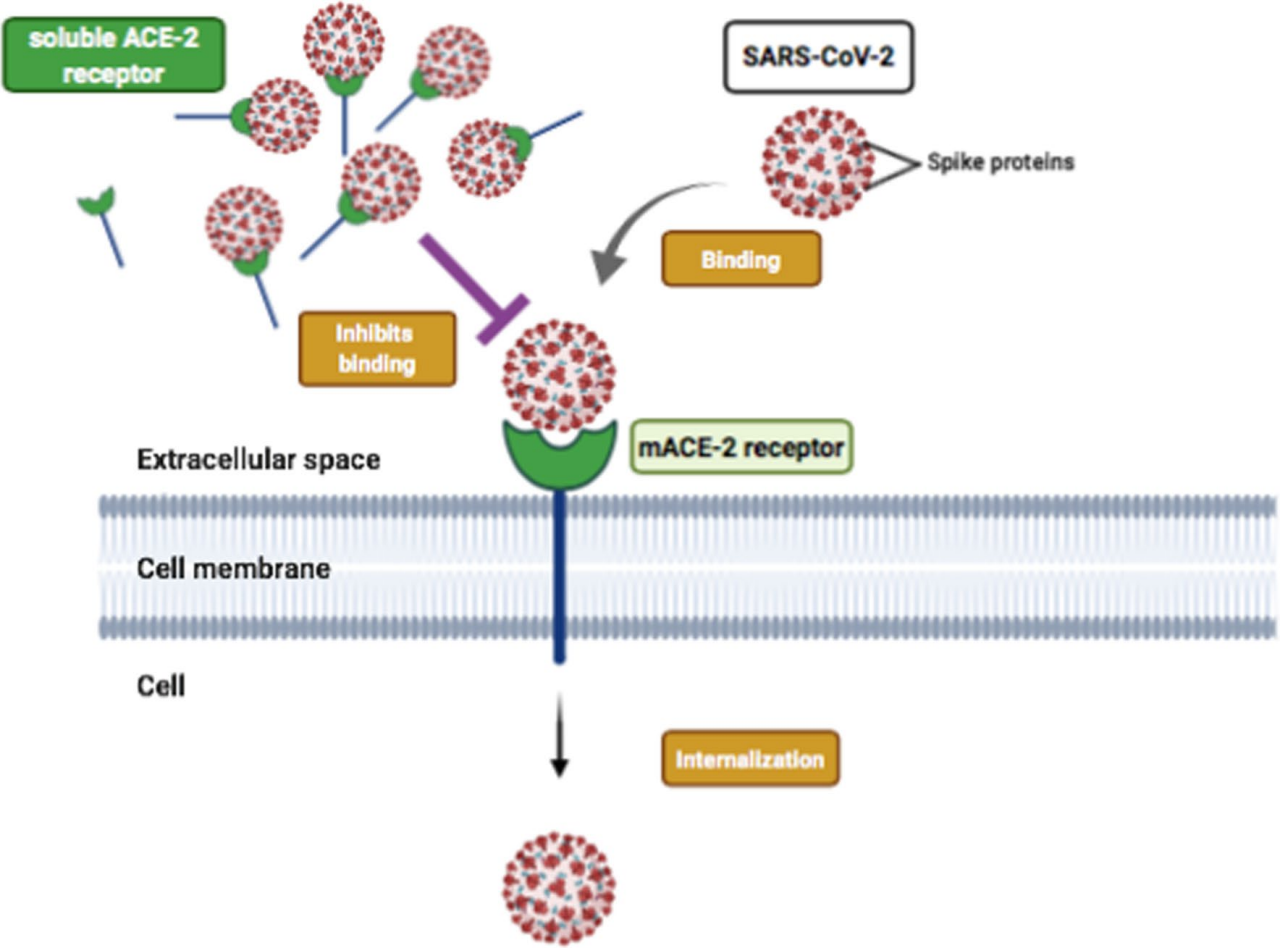
Membrane bound ACE-2 receptor binds to the spike proteins on SARS-CoV-2, which helps in its internalization. However, soluble form of this receptor (sACE-2) acts a scavenger to prevent the internalization of virus by inhibiting the binding process

Many receptors that participate in the entry of viruses in cells, rather than evoking an immune response against the viral particles that happens in the case of SRs, facilitate the replication and dissemination of the pathogen. One of such receptors is Angiotensin-converting Enzyme 2 ACE-2. It is a transmembrane metallopeptidase that functions as a monocarboxypeptidase to cleave diverse regulatory peptides such at the carboxyl-terminal of Angiotensins II and I, bradykinin, kinetensin, and neurotensin [127, 128]. It functions as a vasodilator, unlike ACE-1. ACE-2 was first isolated from Vero E6 (African green monkey kidney cell line) that interact with the S1 domain of the SARS-CoV (Coronavirus) S protein through S1-Ig interaction. ACE-2 permits the cell–cell fusion and then replication of the virus that was confirmed when the soluble form of ACE-2 inhibited the S1-Ig interactions with Vero E6 cells and ACE2-transfected 293 T cells shows signs of cytopathicity on infection respectively. ACE-2 is a principal receptor for SARS-CoV was also confirmed *in* vivo on established mouse animal models [129, 130]. A novel virus, SARS-CoV-2 originated from bats and pangolins as possible intermediate hosts showed structural homology, 76.5% identity in amino acid sequences of spike proteins with SARS-CoV [131]. Also, the spike proteins of SARS-CoV-2 recognize and bind to human ACE-2 with high affinity than SARS-CoV thus contributing to a higher rate of transmission [132]. Overexpression of membrane-bound ACE-2 on HeLa cells from diverse species like humans, civets, pigs, other than mouse show SARS-Cov-2 uses only ACE-2 receptor for entry and not other receptors like aminopeptidase N and dipeptidyl peptidase 4 [133]. Of note, however, most of the scavenger receptors discussed above have both membrane-bound forms and soluble forms that participate in scavenging. But ACE-2 only in its soluble form might participate in preventing the viral entry and replication unlike its membrane form, which is one of the properties of scavenger receptors (Fig. 7). It is suggested that free ACE-2 competitively binds to the spike proteins of CoV-2 thus preventing the virus to bind ACE-2 found excessively on alveolar epithelial type II and hence the viral spread. Also, it prevents lung injury by negatively regulating the Renin-Angiotensin pathway (RAS) by increasing the ratio of ACE/ACE-2 [134]. Thus, classifying ACE-2 as a prospective scavenger receptor will be assessed if additional information becomes available.

## Conclusions

Initially, the role of scavenger receptors was confined to their involvement in lipoprotein binding. With time it was clear just like other PPRs, SRs have dynamically complex interactions with an extraordinary repertoire of ligands like PAMPs, DAPMs, modified self-molecules etc. This versatility is because this family of receptors is classified in various classes each with diverse functional roles in host–pathogen interactions, innate immunity, adaptive immune response, inflammation signaling, ligand delivery and antigen presentation etc. Also, different SRs can bind to the same type of ligands and a single SR can show interaction with a variety of pathogenic ligands. Another important property that enables these receptors to have a dynamic behaviour is to have a reversible interaction with various co-receptors in response to ligands thus taking part in homeostasis and in combatting infections. Interestingly, each receptor type can induce inflammation to control infection under some conditions while having an anti-inflammatory response in some other conditions. But mostly SRs are capable to contribute to pathogen elimination by controlling the recruitment and the activation of phagocytic cells and regulating inflammatory response through proinflammatory cytokine production. Differential responses generated by a single receptor against different ligands and with various co-receptors need to be comprehensively studied. In our study, we conclude SCARB1 (Class B receptor) to play a vital role in the *E.coli*-induced activation of TLR4 signaling cascades thus providing a deeper insight into host pathogen interactions. Therapeutic tools involving functional manipulation of these receptors through various approaches are attractive prospects to explore their role as therapeutic targets in inflammatory diseases and present an opportunity for the development of clinical therapies to target autoimmunity. Also, sophisticated techniques like proteomics, transcriptomic approaches, biophysical methods and super-resolution techniques are required to understand the signalling complexes and clusters in inflammation signalling pathways and the biology of scavenger receptors overall.

## Data Availability

Not applicable.

## Abbreviations

LDLs: Low-density lipoproteins
DAMPS: Damage-associated molecular patterns
PAMPs: Pathogen-associated molecular patterns
LPS: Lipopolysaccharide and
LTA: Lipoteichoic acid
PRRs: Pattern recognition receptors
TLRs: Toll like receptors
CLEC: C-type lectin domain
HSV-1: Herpes simplex virus type 1
CDE: Clathrin-dependent endocytosis
CIE: Clathrin-independent endocytosis
ROS: Reactive oxygen species
HFMD: Hand Foot and Mouth Disease
STST3: Signal transducer and activator of transcription 3
SRCL: Scavenger receptor with C-type lectin
MAPKs: Mitogen-activated protein kinases
HCV: Hepatitis C virus
DCs: Dendritic cells
BECs: Bronchial epithelial cells
PGMECs: Primary goat mammary epithelial cells
LIMP-2: Lysosome membrane protein 2
EV71: Enterovirus 71
LAMP: Lysosome-associated membrane glycoprotein
LOX-1: Low-density lipoprotein receptor
CHO-K1: Chinese hamster ovary-K1
ASGPR1: Asialoglycoprotein receptor 1
EGF: Epidermal growth factor
CXCL16: Chemokine 16
SR-PSOX: Scavenger receptor for phosphatidylserine and oxidized LDL
CXCR6: CXC-chemokine receptor 6
ADAM10: A Disintegrin and metalloproteinase domain-containing protein 10
FAS1: Fasciclin 1
TWEAK: TNF‐related weak inducer of apoptosis
RAGE: Receptor for advanced glycation end products
HA: Hyaluronan
GAS: Group A *Streptococcus*
LRP1: LDLR-related protein 1
ACE-2: Angiotensin-converting Enzyme 2
SARS-CoV: Severe acute respiratory syndrome coronavirus
RAS: Renin Angiotensin pathway

## References

[1] Brown MS, Goldstein JL (1983). Lipoprotein metabolism in the macrophage: implications for cholesterol deposition in atherosclerosis. Annu Rev Biochem.

[2] Pluddemann A, Mukhopadhyay S, Gordon S (2011). Innate immunity to intracellular pathogens: macrophage recep- tors and responses to microbial entry. Immunol Rev.

[3] Kishore U (2009). Target pattern recognition in innate immunity. Preface Adv Exp Med Biol.

[4] Areschoug T, Gordon S (2009). Scavenger receptors: role in innate immunity and microbial pathogenesis. Cell Microbiol.

[5] Plüddemann A, Neyen C, Gordon S. Macrophage scavenger receptors and host-derived ligands. Methods. 2007 10.1016/j.ymeth.2007.06.004 17920517

[6] Gordon S (2002). Pattern recognition receptors: doubling up for the innate immune response. Cell.

[7] Mukhopadhyay S, Gordon S (2004). The role of scavenger receptors in pathogen recognition and innate immunity. Immunobiology.

[8] Murphy JE, Tedbury PR, Homer-Vanniasinkam S, Walker JH, Ponnambalam S (2005). Biochemistry and cell biology of mammalian scavenger receptors. Atherosclerosis.

[9] Areschoug T, Gordon S (2008). Pattern recognition receptors and their role in innate immunity: focus on microbial protein ligands. Trends Innate Immun.

[10] Zani IA, Stephen SL, Mughal NA, Russell D, Homer-Vanniasinkam S, Wheatcroft SB (2015). Scavenger receptor structure and function in health and disease. Cells.

[11] PrabhuDas MR, Baldwin CL, Bollyky PL, Bowdish DM, Drickamer K, Febbraio M, Herz J, Kobzik L, Krieger M, Loike J, McVicker B (2017). A consensus definitive classification of scavenger receptors and their roles in health and disease. J Immunol Res.

[12] Krieger M (1997). The other side of scavenger receptors: pattern recognition for host defense. Curr Opin Lipidol.

[13] Yu H, Ha T, Liu L, Wang X, Gao M, Kelley J (2012). Scavenger receptor A (SR-A) is required for LPS-induced TLR4 mediated NF-κB activation in macrophages. Biochim Biophys Acta.

[14] Hampton RY, Golenbock DT, Penman M, Krieger M, Raetz CR (1999). Recognition and plasma clearance of endotoxin by scavenger receptors. Nature.

[15] Dunne DW, Resnick D, Greenberg J, Krieger M, Joiner KA (1994). The type I macrophage scavenger receptor binds to gram-positive bacteria and recognizes lipoteichoic acid. Proc Natl Acad Sci.

[16] Peiser L, Makepeace K, Plüddemann A, Savino S, Wright JC, Pizza M (2006). Identification of Neisseria meningitidis nonlipopolysaccharide ligands for class A macrophage scavenger receptor by using a novel assay. Infect Immun.

[17] Thomas CA, Li Y, Kodama T, Suzuki H, Silverstein SC, El Khoury J (2000). Protection from lethal Gram-positive infection by macrophage scavenger receptor–dependent phagocytosis. J Exp Med.

[18] Arredouani MS, Yang Z, Imrich A, Ning Y, Qin G, Kobzik L (2006). The macrophage scavenger receptor SR-AI/II and lung defense against pneumococci and particles. Am J Respir Cell Mol Biol.

[19] Ojala JR, Pikkarainen T, Tuuttila A, Sandalova T, Tryggvason K (2007). Crystal structure of the cysteine-rich domain of scavenger receptor MARCO reveals the presence of a basic and an acidic cluster that both contribute to ligand recognition. J Biol Chem.

[20] Plüddemann A, Mukhopadhyay S, Sankala M, Savino S, Pizza M, Rappuoli R (2009). SR-A, MARCO and TLRs differentially recognise selected surface proteins from Neisseria meningitidis: an example of fine specificity in microbial ligand recognition by innate immune receptors. J Innate Immun.

[21] Fischer N, Haug M, Kwok WW, Kalbacher H, Wernet D, Dannecker GE (2010). Involvement of CD91 and scavenger receptors in Hsp70 facilitated activation of human antigen specific CD4+ memory T cells. Eur J Immunol.

[22] Amiel E, Alonso A, Uematsu S, Akira S, Poynter ME, Berwin B (2009). Pivotal Advance: toll like receptor regulation of scavenger receptor A mediated phagocytosis. J Leukoc Biol.

[23] Bowdish DM, Sakamoto K, Kim MJ, Kroos M, Mukhopadhyay S, Leifer CA, et al. MARCO, TLR2, and CD14 are required for macrophage cytokine responses to mycobacterial trehalose dimycolate and Mycobacterium tuberculosis. PLoS Pathog. 2009;5. 10.1371/journal.ppat.1000474 PMC2688075 19521507

[24] MacLeod DT, Nakatsuji T, Yamasaki K, Kobzik L, Gallo RL (2013). HSV-1 exploits the innate immune scavenger receptor MARCO to enhance epithelial adsorption and infection. Nat Commun.

[25] Zizzo G, Hilliard BA, Monestier M, Cohen PL (2012). Efficient clearance of early apoptotic cells by human macrophages requires M2c polarization and MerTK induction. J Immunol.

[26] Tian Y, Zhou K, Hu J, Shan MF, Chen HJ, Cheng S, et al. Scavenger receptor class a, member 3 is associated with severity of hand, foot, and mouth disease in a case-control study. Medicine. 2019;98. 10.1097/MD.0000000000017471 PMC6783241 31577778

[27] Ohtani K, Suzuki Y, Eda S, Kawai T, Kase T, Keshi H (2001). The membrane-type collectin CL-P1 is a scavenger receptor on vascular endothelial cells. J Biol Chem.

[28] Jang S, Ohtani K, Fukuoh A, Yoshizaki T, Fukuda M, Motomura W (2009). Scavenger receptor collectin placenta 1 (CL-P1) predominantly mediates zymosan phagocytosis by human vascular endothelial cells. J Biol Chem.

[29] Li JY, Paragas N, Ned RM, Qiu A, Viltard M, Leete T (2009). Scara5 is a ferritin receptor mediating non-transferrin iron delivery. Dev Cell.

[30] Yan N, Zhang S, Yang Y, Cheng L, Li C, Dai L (2012). Therapeutic upregulation of Class A scavenger receptor member 5 inhibits tumor growth and metastasis. Cancer Sci.

[31] Bull HA, Brickell PM, Dowd PM (1994). Src related protein tyrosine kinases are physically associated with the surface antigen CD36 in human dermal microvascular endothelial cells. FEBS Lett.

[32] Stewart CR, Stuart LM, Wilkinson K, Van Gils JM, Deng J, Halle A (2010). CD36 ligands promote sterile inflammation through assembly of a Toll-like receptor 4 and 6 heterodimer. Nat Immunol.

[33] Stuart LM, Deng J, Silver JM, Takahashi K, Tseng AA, Hennessy EJ (2005). Response to *Staphylococcus aureus* requires CD36-mediated phagocytosis triggered by the COOH-terminal cytoplasmic domain. J Cell Biol.

[34] Vishnyakova TG, Bocharov AV, Baranova IN, Chen Z, Remaley AT, Csako G (2003). Binding and internalization of lipopolysaccharide by Cla-1, a human orthologue of rodent scavenger receptor B1. J Biol Chem.

[35] Baranova IN, Vishnyakova TG, Bocharov AV, Leelahavanichkul A, Kurlander R, Chen Z (2012). Class B scavenger receptor types I and II and CD36 mediate bacterial recognition and proinflammatory signaling induced by *Escherichia coli*, lipopolysaccharide, and cytosolic chaperonin 60. J Immunol.

[36] Guo L, Zheng Z, Ai J, Huang B, Li XA (2014). Hepatic scavenger receptor BI protects against polymicrobial-induced sepsis through promoting LPS clearance in mice. J Biol Chem.

[37] Barth H, Schnober EK, Neumann-Haefelin C, Thumann C, Zeisel MB, Diepolder HM (2008). Scavenger receptor class B is required for hepatitis C virus uptake and cross-presentation by human dendritic cells. J Virol.

[38] Dieudonné A, Torres D, Blanchard S, Taront S, Jeannin P, Delneste Y, et al. Scavenger receptors in human airway epithelial cells: role in response to double-stranded RNA. PLoS ONE. 2012;7. 10.1371/journal.pone.0041952 PMC3413698 22879901

[39] Yalaoui S, Huby T, Franetich JF, Gego A, Rametti A, Moreau M (2008). Scavenger receptor BI boosts hepatocyte permissiveness to Plasmodium infection. Cell Host Microbe.

[40] Schäfer G, Guler R, Murray G, Brombacher F, Brown GD. The role of scavenger receptor B1 in infection with Mycobacterium tuberculosis in a murine model. PLoS ONE. 2009;4. 10.1371/journal.pone.0008448 PMC2794535 20041149

[41] Gabriel C, Becher-Deichsel A, Hlavaty J, Mair G, Walter I (2016). The physiological expression of scavenger receptor SR-B1 in canine endometrial and placental epithelial cells and its potential involvement in pathogenesis of pyometra. Theriogenology.

[42] Baranova IN, Kurlander R, Bocharov AV, Vishnyakova TG, Chen Z, Remaley AT (2008). Role of human CD36 in bacterial recognition, phagocytosis, and pathogen-induced JNK-mediated signaling. J Immunol.

[43] Means TK, Mylonakis E, Tampakakis E, Colvin RA, Seung E, Puckett L (2009). Evolutionarily conserved recognition and innate immunity to fungal pathogens by the scavenger receptors SCARF1 and CD36. J Exp Med.

[44] Hawkes M, Li X, Crockett M, Diassiti A, Finney C, Min-Oo G (2010). CD36 deficiency attenuates experimental mycobacterial infection. BMC Infect Dis.

[45] Cao D, Luo J, Chen D, Xu H, Shi H, Jing X (2016). CD36 regulates lipopolysaccharide-induced signaling pathways and mediates the internalization of Escherichia coli in cooperation with TLR4 in goat mammary gland epithelial cells. Sci Rep.

[46] Yamayoshi S, Yamashita Y, Li J, Hanagata N, Minowa T, Takemura T (2009). Scavenger receptor B2 is a cellular receptor for enterovirus 71. Nat Med.

[47] Yamayoshi S, Iizuka S, Yamashita T, Minagawa H, Mizuta K, Okamoto M (2012). Human SCARB2-dependent infection by coxsackievirus A7, A14, and A16 and enterovirus 71. J Virol.

[48] Yamayoshi S, Ohka S, Fujii K, Koike S (2013). Functional comparison of SCARB2 and PSGL1 as receptors for enterovirus 71. J Virol.

[49] Ramet M, Pearson A, Manfruelli P, Li X, Koziel H, Gobel V (2001). Drosophila scavenger receptor CI is a pattern recognition receptor for bacteria. Immunity.

[50] Song L, Lee C, Schindler C (2011). Deletion of the murine scavenger receptor CD68. J Lipid Res.

[51] Amanzada A, Malik IA, Blaschke M, Khan S, Rahman H, Ramadori G (2013). Identification of CD68(+) neutrophil granulocytes in in vitro model of acute inflammation and inflammatory bowel disease. Int J Clin Exp Pathol.

[52] Barros MH, Hauck F, Dreyer JH, Kempkes B, Niedobitek G (2013). Macrophage polarization: an immunohistochemical approach for identifying M1 and M2 macrophages. PLoS ONE.

[53] Papageorgiou IE, Lewen A, Galow LV, Cesetti T, Scheffel J, Regen T (2016). TLR4-activated microglia require IFN-γ to induce severe neuronal dysfunction and death in situ. Proc Natl Acad Sci USA.

[54] Cha SJ, Park K, Srinivasan P, Schindler CW, Van Rooijen N, Stins M (2015). CD68 acts as a major gateway for malaria sporozoite liver infection. J Exp Med.

[55] Lin XP, Almqvist N, Telemo E (2005). Human small intestinal epithelial cells constitutively express the key elements for antigen processing and the production of exosomes. Blood Cells Mol Dis.

[56] Wu Z, Sawamura T, Kurdowska AK, Ji HL, Idell S, Fu J (2011). LOX-1 deletion improves neutrophil responses, enhances bacterial clearance, and reduces lung injury in a murine polymicrobial sepsis model. Infect Immun.

[57] Tanigawa H, Miura SI, Matsuo Y, Fujino M, Sawamura T, Saku K (2006). Dominant-negative lox-1 blocks homodimerization of wild-type Lox-1–induced cell proliferation through extracellular signal regulated kinase 1/2 activation. Hypertension.

[58] Khaidakov M, Wang X, Mehta JL. Potential involvement of LOX-1 in functional consequences of endothelial senescence. PLoS ONE. 2011;6. 10.1371/journal.pone.0020964 PMC3115962 21698300

[59] Shimaoka T, Kume N, Minami M, Hayashida K, Sawamura T, Kita T (2001). LOX-1 supports adhesion of Gram-positive and Gram-negative bacteria. J Immunol.

[60] Jeannin P, Bottazzi B, Sironi M, Doni A, Rusnati M, Presta M (2005). Complexity and complementarity of outer membrane protein A recognition by cellular and humoral innate immunity receptors. Immunity.

[61] Herre J, Willment JA, Gordon S, Brown GD. The role of Dectin-1 in antifungal immunity. Crit Rev Immunol. 2004;24. 10.1615/critrevimmunol.v24.i3.30 15482254

[62] Drummond RA, Brown GD (2011). The role of Dectin-1 in the host defence against fungal infections. Curr Opin Microbiol.

[63] Reid DM, Montoya M, Taylor PR, Borrow P, Gordon S, Brown GD, Wong SY (2004). Expression of the β glucan receptor, Dectin 1, on murine leukocytes in situ correlates with its function in pathogen recognition and reveals potential roles in leukocyte interactions. J Leukoc Biol.

[64] Chen SM, Shen H, Zhang T, Huang X, Liu XQ, Guo SY (2017). Dectin-1 plays an important role in host defense against systemic Candida glabrata infection. Virulence.

[65] Azad AK, Rajaram MV, Schlesinger LS. Exploitation of the macrophage mannose receptor (CD206) in infectious disease diagnostics and therapeutics. J Cytol Mol Biol. 2014;1(1). 10.13188/2325-4653.1000003 PMC3963702 24672807

[66] Wollenberg A, Oppel T, Schottdorf EM, Günther S, Moderer M, Mommaas M (2002). Expression and function of the mannose receptor CD206 on epidermal dendritic cells in inflammatory skin diseases. J Invest Dermatol..

[67] Kaku Y, Imaoka H, Morimatsu Y, Komohara Y, Ohnishi K, Oda H, et al. Overexpression of CD163, CD204 and CD206 on alveolar macrophages in the lungs of patients with severe chronic obstructive pulmonary disease. PLoS ONE. 2014;9. 10.1371/journal.pone.0087400 PMC3907529 24498098

[68] Dai K, Huang L, Sun X, Yang L, Gong Z (2015). Hepatic CD206 positive macrophages express amphiregulin to promote the immunosuppressive activity of regulatory T cells in HBV infection. J Leukoc Biol.

[69] Guy CS, Rankin SL, Michalak TI (2011). Hepatocyte cytotoxicity is facilitated by asialoglycoprotein receptor. Hepatology.

[70] McVicker BL, Thiele GM, Casey CA, Osna NA, Tuma DJ (2013). Susceptibility to T cell-mediated liver injury is enhanced in asialoglycoprotein receptor-deficient mice. Int Immunopharmacol.

[71] Zhang L, Tian Y, Wen Z, Zhang F, Qi Y, Huang W (2016). Asialoglycoprotein receptor facilitates infection of PLC/PRF/5 cells by HEV through interaction with ORF2. J Med Virol.

[72] Beauvillain C, Meloni F, Sirard JC, Blanchard S, Jarry U, Scotet M (2010). The scavenger receptors SRA-1 and SREC-I cooperate with TLR2 in the recognition of the hepatitis C virus non-structural protein 3 by dendritic cells. J Hepatol.

[73] Murshid A, Gong J, Prince T, Borges TJ, Calderwood SK. Scavenger receptor SREC-I mediated entry of TLR4 into lipid microdomains and triggered inflammatory cytokine release in RAW 264.7 cells upon LPS activation. PLoS ONE. 2015;10. 10.1371/journal.pone.0122529 PMC4383338 25836976

[74] Ramirez-Ortiz ZG, Pendergraft WF, Prasad A, Byrne MH, Iram T, Blanchette CJ (2013). The scavenger receptor SCARF1 mediates the clearance of apoptotic cells and prevents autoimmunity. Nat Immunol.

[75] Ishii J, Adachi H, Aoki J, Koizumi H, Tomita S, Suzuki T (2002). SREC-II, a new member of the scavenger receptor type F family, trans-interacts with SREC-I through its extracellular domain. J Biol Chem.

[76] Iram T, Ramirez-Ortiz Z, Byrne MH, Coleman UA, Kingery ND, Means TK (2016). Megf10 is a receptor for C1Q that mediates clearance of apoptotic cells by astrocytes. J Neurosci.

[77] Morizawa YM, Hirayama Y, Ohno N, Shibata S, Shigetomi E, Sui Y (2017). Reactive astrocytes function as phagocytes after brain ischemia via ABCA1-mediated pathway. Nat Commun.

[78] Ziegenfuss JS, Biswas R, Avery MA, Hong K, Sheehan AE, Yeung YG (2008). Draper-dependent glial phagocytic activity is mediated by Src and Syk family kinase signalling. Nature.

[79] Zhou Z, Caron E, Hartwieg E, Hall A, Horvitz HR. The C. elegans PH domain protein CED-12 regulates cytoskeletal reorganization via a Rho/Rac GTPase signaling pathway. Developmental cell. 2001;1(4):477–89. 10.1016/s1534-5807(01)00058-2 11703939

[80] Chung WS, Clarke LE, Wang GX, Stafford BK, Sher A, Chakraborty C (2013). Astrocytes mediate synapse elimination through MEGF10 and MERTK pathways. Nature.

[81] Tabata S, Kadowaki N, Kitawaki T, Shimaoka T, Yonehara S, Yoshie O (2005). Distribution and kinetics of SR-PSOX/CXCL16 and CXCR6 expression on human dendritic cell subsets and CD4+ T cells. J Leukoc Biol.

[82] Hundhausen C, Schulte A, Schulz B, Andrzejewski MG, Schwarz N, Von Hundelshausen P (2007). Regulated shedding of transmembrane chemokines by the disintegrin and metalloproteinase 10 facilitates detachment of adherent leukocytes. J Immunol.

[83] Uza N, Nakase H, Yamamoto S, Yoshino T, Takeda Y, Ueno S (2011). SR-PSOX/CXCL16 plays a critical role in the progression of colonic inflammation. Gut.

[84] Shimaoka T, Seino KI, Kume N, Minami M, Nishime C, Suematsu M (2007). Critical role for CXC chemokine ligand 16 (SR-PSOX) in Th1 response mediated by NKT cells. J Immunol.

[85] Shimaoka T, Nakayama T, Fukumoto N, Kume N, Takahashi S, Yamaguchi J (2004). Cell surface anchored SR PSOX/CXC chemokine ligand 16 mediates firm adhesion of CXC chemokine receptor 6 expressing cells. J Leukoc Biol.

[86] Hudspeth K, Donadon M, Cimino M, Pontarini E, Tentorio P, Preti M (2016). Human liver-resident CD56bright/CD16neg NK cells are retained within hepatic sinusoids via the engagement of CCR5 and CXCR6 pathways. J Autoimmun.

[87] Irjala H, Elima K, Johansson EL, Merinen M, Kontula K, Alanen K (2003). The same endothelial receptor controls lymphocyte traffic both in vascular and lymphatic vessels. Eur J Immunol.

[88] Karikoski M, Irjala H, Maksimow M, Miiluniemi M, Granfors K, Hernesniemi S (2009). Clever-1/Stabilin-1 regulates lymphocyte migration within lymphatics and leukocyte entrance to sites of inflammation. Eur J Immunol.

[89] Adachi H, Tsujimoto M (2002). FEEL-1, a novel scavenger receptor with in vitro bacteria-binding and angiogenesis-modulating activities. J Biol Chem.

[90] Jung MY, Park SY, Kim IS (2007). Stabilin 2 is involved in lymphocyte adhesion to the hepatic sinusoidal endothelium via the interaction with αMβ2 integrin. J Leukoc Biol.

[91] Kim S, Bae D-J, Hong M, Park S-Y, Kim I-S (2010). The conserved histidine in epidermal growth factor-like domains of stabilin-2 modulates pH-dependent recognition of phosphatidylserine in apoptotic cells. Int J Biochem Cell Biol.

[92] Buechler C, Ritter M, Orsó E, Langmann T, Klucken J, Schmitz G (2000). Regulation of scavenger receptor CD163 expression in human monocytes and macrophages by pro and antiinflammatory stimuli. J Leukoc Biol.

[93] Etzerodt A, Moestrup SK (2013). CD163 and inflammation: biological, diagnostic, and therapeutic aspects. Antioxid Redox Signal.

[94] Canton J, Neculai D, Grinstein S (2013). Scavenger receptors in homeostasis and immunity. Nat Rev Immunol.

[95] Philippidis P, Mason JC, Evans BJ, Nadra I, Taylor KM, Haskard DO (2004). Hemoglobin scavenger receptor CD163 mediates interleukin-10 release and heme oxygenase-1 synthesis: antiinflammatory monocyte-macrophage responses in vitro, in resolving skin blisters in vivo, and after cardiopulmonary bypass surgery. Circ Res.

[96] Moeller JB, Nielsen MJ, Reichhardt MP, Schlosser A, Sorensen GL, Nielsen O (2012). CD163-L1 is an endocytic macrophage protein strongly regulated by mediators in the inflammatory response. J Immunol.

[97] González-Domínguez É, Samaniego R, Flores-Sevilla JL, Campos-Campos SF, Gómez-Campos G, Salas A (2015). CD163L1 and CLEC5A discriminate subsets of human resident and inflammatory macrophages in vivo. J Leukoc Biol.

[98] Holm D, Fink DR, Steffensen MA, Schlosser A, Nielsen O, Moeller JB (2013). Characterization of a novel human scavenger receptor cysteine-rich molecule SCART1 expressed by lymphocytes. Immunobiology.

[99] Telfer JC, Baldwin CL. Baldwin. Bovine gamma delta T cells and the function of gamma delta T cell specific WC1 co-receptors. Cell Immunol. 2015;296:76–86. 10.1016/j.cellimm.2015.05.003 26008759

[100] Vera J, Fenutría R, Cañadas O, Figueras M, Mota R, Sarrias MR (2009). The CD5 ectodomain interacts with conserved fungal cell wall components and protects from zymosan-induced septic shock-like syndrome. Proc Natl Acad Sci.

[101] Braun M, Müller B, Ter Meer D, Raffegerst S, Simm B, Wilde S (2011). The CD6 scavenger receptor is differentially expressed on a CD56dim natural killer cell subpopulation and contributes to natural killer-derived cytokine and chemokine secretion. J Innate Immun.

[102] Sarrias MR, Farnós M, Mota R, Sánchez-Barbero F, Ibáñez A, Gimferrer I (2007). CD6 binds to pathogen-associated molecular patterns and protects from LPS-induced septic shock. Proc Natl Acad Sci.

[103] Ibrahim ZA, Armour CL, Phipps S, Sukkar MB (2013). RAGE and TLRs: relatives, friends or neighbours?. Mol Immunol.

[104] Sakaguchi M, Murata H, Yamamoto KI, Ono T, Sakaguchi Y, Motoyama A, et al. TIRAP, an adaptor protein for TLR2/4, transduces a signal from RAGE phosphorylated upon ligand binding. PloS One. 2011;6. 10.1371/journal.pone.0023132 PMC3148248 21829704

[105] Qin YH, Dai SM, Tang GS, Zhang J, Ren D, Wang ZW (2009). HMGB1 enhances the proinflammatory activity of lipopolysaccharide by promoting the phosphorylation of MAPK p38 through receptor for advanced glycation end products. J Immunol.

[106] Xu D, Young J, Song D, Esko JD (2011). Heparan sulfate is essential for high mobility group protein 1 (HMGB1) signaling by the receptor for advanced glycation end products (RAGE). J Biol Chem.

[107] Ghavami S, Rashedi I, Dattilo BM, Eshraghi M, Chazin WJ, Hashemi M (2008). S100A8/A9 at low concentration promotes tumor cell growth via RAGE ligation and MAP kinase-dependent pathway. J Leukoc Biol.

[108] Schelbergen RF, Blom AB, van den Bosch MH, Slöetjes A, Abdollahi-Roodsaz S, Schreurs BW (2012). Alarmins S100A8 and S100A9 elicit a catabolic effect in human osteoarthritic chondrocytes that is dependent on Toll-like receptor 4. Arthritis Rheum.

[109] Ichikawa M, Williams R, Wang L, Vogl T, Srikrishna G (2011). S100A8/A9 activate key genes and pathways in colon tumor progression. Mol Cancer Res.

[110] Yamamoto Y, Harashima A, Saito H, Tsuneyama K, Munesue S, Motoyoshi S (2011). Septic shock is associated with receptor for advanced glycation end products ligation of LPS. J Immunol.

[111] Bangert A, Andrassy M, Müller AM, Bockstahler M, Fischer A, Volz CH (2016). Critical role of RAGE and HMGB1 in inflammatory heart disease. Proc Natl Acad Sci.

[112] Nakamura H, Suenaga N, Taniwaki K, Matsuki H, Yonezawa K, Fujii M (2004). Constitutive and induced CD44 shedding by ADAM-like proteases and membrane-type 1 matrix metalloproteinase. Cancer Res.

[113] Liang J, Jiang D, Griffith J, Yu S, Fan J, Zhao X (2007). CD44 is a negative regulator of acute pulmonary inflammation and lipopolysaccharide-TLR signaling in mouse macrophages. J Immunol.

[114] Kawana H, Karaki H, Higashi M, Miyazaki M, Hilberg F, Kitagawa M (2008). CD44 suppresses TLR-mediated inflammation. J Immunol.

[115] Muto J, Yamasaki K, Taylor KR, Gallo RL (2009). Engagement of CD44 by hyaluronan suppresses TLR4 signaling and the septic response to LPS. Mol Immunol.

[116] Qadri M, Almadani S, Jay GD, Elsaid KA (2018). Role of CD44 in regulating TLR2 activation of human macrophages and downstream expression of proinflammatory cytokines. J Immunol.

[117] Schommer NN, Muto J, Nizet V, Gallo RL (2014). Hyaluronan breakdown contributes to immune defense against group A Streptococcus. J Biol Chem.

[118] Khurana SS, Riehl TE, Moore BD, Fassan M, Rugge M, Romero-Gallo J (2013). The hyaluronic acid receptor CD44 coordinates normal and metaplastic gastric epithelial progenitor cell proliferation. J Biol Chem.

[119] van der Windt GJ, Florquin S, De Vos AF, Van’t Veer C, Queiroz KC, Liang J, et al. CD44 deficiency is associated with increased bacterial clearance but enhanced lung inflammation during Gram-negative pneumonia. Am J Pathol. 2010;177:2483–94. 10.2353/ajpath.2010.100562 PMC2966805 20864681

[120] Li P, Fujimoto K, Bourguingnon L, Yukl S, Deeks S, Wong JK (2014). Exogenous and endogenous hyaluronic acid reduces HIV infection of CD4(+) T cells. Immunol Cell Biol.

[121] Abe T, Fukuhara T, Wen X, Ninomiya A, Moriishi K, Maehara Y (2012). CD44 participates in IP-10 induction in cells in which hepatitis C virus RNA is replicating, through an interaction with toll-like receptor 2 and hyaluronan. J Virol.

[122] Presicce P, Giannelli S, Taddeo A, Villa ML, Della BS (2009). Human defensins activate monocyte-derived dendritic cells, promote the production of proinflammatory cytokines, and up-regulate the surface expression of CD91. J Leukoc Biol.

[123] Pawaria S, Binder RJ (2011). CD91-dependent programming of T-helper cell responses following heat shock protein immunization. Nat Commun.

[124] Basu S, Binder RJ, Suto R, Anderson KM, Srivastava PK (2000). Necrotic but not apoptotic cell death releases heat shock proteins, which deliver a partial maturation signal to dendritic cells and activate the NF-kappa B pathway. Int Immunol.

[125] Sedlackova L, Nguyen TT, Zlacka D, Sosna A, Hromadnikova I (2009). Cell surface and relative mRNA expression of heat shock protein 70 in human synovial cells. Autoimmunity.

[126] Bartolome F, Antequera D, Tavares E, Pascual C, Maldonado R, Camins A (2017). Obesity and neuroinflammatory phenotype in mice lacking endothelial megalin. J Neuroinflamm.

[127] Donoghue M, Hsieh F, Baronas E, Godbout K, Gosselin M, Stagliano N (2000). Acton, A novel angiotensin-converting enzyme-related carboxypeptidase (ACE2) converts angiotensin I to angiotensin 1–9. Circ Res.

[128] Warner FJ, Smith AI, Hooper NM, Turner AJ (2004). Angiotensin- converting enzyme-2: a molecular and cellular perspective. Cell Mol Life Sci.

[129] Kuba K, Imai Y, Rao S, Gao H, Guo F, Guan B (2005). A crucial role of angiotensin converting enzyme 2 (ACE2) in SARS coronavirus—induced lung injury. Nat Med.

[130] Yang XH, Deng W, Tong Z, Liu YX, Zhang LF, Zhu H (2007). Mice transgenic for human angiotensin-converting enzyme 2 provide a model for SARS coronavirus infection. Comp Med.

[131] Xu X, Chen P, Wang J, Feng J, Zhou H, Li X (2020). Evolution of the novel coronavirus from the ongoing Wuhan outbreak and modeling of its Spike protein for risk of human transmission. Sci China Life Sci.

[132] Wan Y, Shang J, Graham R, Baric RS, Li F. Receptor recognition by novel coronavirus from Wuhan: an analysis based on decade-long structural studies of SARS. J Virol. 2020;94. 10.1128/JVI.00127-20 PMC7081895 31996437

[133] Zhou P, Yang XL, Wang XG, Hu B, Zhang L, Zhang W (2020). A pneumonia outbreak associated with a new coronavirus of probable bat origin. Nature.

[134] Yu L, Yuan K, Phuong HT, Park BM, Kim SH (2016). Angiotensin-(1–5), an active mediator of renin-angiotensin system, stimulates ANP secretion via Mas receptor. Peptides.

